# Applications of artificial intelligence and computational approaches to imaging for hypertension identification, phenotyping, and outcome prediction: a systematic review

**DOI:** 10.1093/ehjdh/ztag063

**Published:** 2026-04-20

**Authors:** Mohanad Alkhodari, Prenali D Sattwika, Hannah R Cutler, George Milner, Turkay Kart, Leontios J Hadjileontiadis, Ahsan H Khandoker, Adam J Lewandowski, Winok Lapidaire, Abhirup Banerjee, Paul Leeson

**Affiliations:** Cardiovascular Clinical Research Facility (CCRF), Division of Cardiovascular Medicine, Radcliffe Department of Medicine, University of Oxford, Oxford, UK; Healthcare Engineering Innovation Group (HEIG), Department of Biomedical Engineering & Biotechnology, Khalifa University, Abu Dhabi, United Arab Emirates; Cardiovascular Clinical Research Facility (CCRF), Division of Cardiovascular Medicine, Radcliffe Department of Medicine, University of Oxford, Oxford, UK; Department of Internal Medicine, Faculty of Medicine, Public Health, and Nursing, Universitas Gadjah Mada, Yogyakarta, Indonesia; Clinical Epidemiology and Biostatistics Unit, Faculty of Medicine, Public Health, and Nursing, Universitas Gadjah Mada, Yogyakarta, Indonesia; Cardiovascular Clinical Research Facility (CCRF), Division of Cardiovascular Medicine, Radcliffe Department of Medicine, University of Oxford, Oxford, UK; Cardiovascular Clinical Research Facility (CCRF), Division of Cardiovascular Medicine, Radcliffe Department of Medicine, University of Oxford, Oxford, UK; Cardiovascular Clinical Research Facility (CCRF), Division of Cardiovascular Medicine, Radcliffe Department of Medicine, University of Oxford, Oxford, UK; Biomedical Image Analysis Group, Department of Computing, Imperial College London, London, UK; Healthcare Engineering Innovation Group (HEIG), Department of Biomedical Engineering & Biotechnology, Khalifa University, Abu Dhabi, United Arab Emirates; Department of Electrical and Computer Engineering, Aristotle University of Thessaloniki, Thessaloniki, Greece; Healthcare Engineering Innovation Group (HEIG), Department of Biomedical Engineering & Biotechnology, Khalifa University, Abu Dhabi, United Arab Emirates; Nuffield Department of Population Health, University of Oxford, Oxford, UK; Cardiovascular Clinical Research Facility (CCRF), Division of Cardiovascular Medicine, Radcliffe Department of Medicine, University of Oxford, Oxford, UK; Institute of Biomedical Engineering, Department of Engineering Science, University of Oxford, Oxford, UK; Cardiovascular Clinical Research Facility (CCRF), Division of Cardiovascular Medicine, Radcliffe Department of Medicine, University of Oxford, Oxford, UK

**Keywords:** Hypertension, Organ damage, Computational machine learning, Medical imaging, Systematic review

## Abstract

Current hypertension guidelines focus on blood pressure control, but incorporating end-organ imaging could improve understanding of disease manifestations. We undertook a systematic review to evaluate current task-level applications of artificial intelligence (AI) and computational approaches to imaging for hypertension identification, phenotyping, and outcome prediction. A systematic search was conducted across multiple databases up to end of December 2025. Retrieved studies were grouped by AI task, and a thematic qualitative analysis per-task was conducted to evaluate organ-specific findings, AI methodologies, and research gaps. For quantitative synthesis, the I2 statistic derived from Cochran’s Q test was used to assess heterogeneity, and forest plots were generated to visualize effect sizes. The review was registered with PROSPERO (CRD42023427430). The search strategy yielded 48 studies. Thematic analysis categorized the studies into five major tasks, with the majority employing supervised learning for classification processes. Nearly half of the studies focused on the heart. However, paucity of studies performed multi-organ assessment, external validation, and phenotyping or predicting future risk. AI and computational approaches in imaging achieved an overall sensitivity of 0.84 [0.69–0.93] in identifying hypertension from normotension, highest with brain imaging. Sensitivity reached 0.92 [0.90–0.94] in discriminating hypertension from hypertrophic cardiomyopathy. Current research focusses primarily on hypertension prediction using single organ information. While results are promising, datasets remain small with limited external validation. There remains a need for discovery-oriented research to uncover disease heterogeneity, multi-organ phenotypes, and support personalized and targeted interventions.

## Graphical Abstract

**Graphical Abstract:**
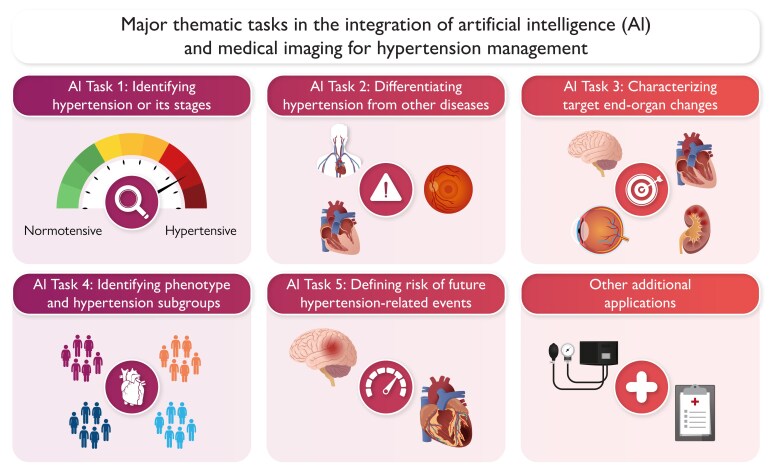

## Introduction

Hypertension is a leading cause of early mortality, affecting over a billion people worldwide.^1^ Current diagnostic and treatment strategies primarily focus on measuring and pharmacologically controlling blood pressure, which may fail to reveal and treat early-stage hypertensive end-organ damage, especially in asymptomatic patients.^2^ Prolonged exposure to hypertension can severely damage organs,^3^ significantly increasing the risk of cardiovascular, cerebral, renal, and other end-organ diseases.^4^ Despite its prevalence, hypertensive organ damage remains under-diagnosed.^5^

Common imaging modalities can detect alterations in organ structure and function. Integration of multi-modal medical imaging into hypertension care could allow a more comprehensive understanding of the impact of hypertension and its progression at an organ level.^6^ However, use of medical imaging has been limited by high costs, limited accessibility and need for expert interpretation.^7^ As a result, imaging is often reserved for identifying secondary causes and detecting organ damage at advanced stages.

Artificial intelligence (AI) and computational approaches have the potential to streamline image interpretation pipelines while enhancing diagnostic precision.^8^ Recent advances enable extraction of complex, often subtle features from organ-based images.^9–11^ They also have the potential to utilize pre-extracted features from images and provide automated, accurate, and cost-effective diagnostic and prognostic support for clinicians.^12^ In hypertension, AI and computational approaches could thereby facilitate earlier detection of structural and functional alterations across multiple organs,^13^ enhancing prevention of adverse complications and supporting therapeutic decision-making. Therefore, this systematic review sought to establish the current state-of-the-art, effectiveness and methodologies underlying AI and computational tools applied to imaging to identify, phenotype, and predict hypertension and its outcomes using organ information.

## Methods

This review was conducted in accordance with the Preferred Reporting Items for Systematic Reviews and Meta-Analyses statement^14^ (see Supplementary material online, Material). The review protocol was registered with PROSPERO ID: CRD42023427430 in June 2023.

### Search strategy and eligibility criteria

The search strategies were developed using multiple electronic databases by combining key terms including ‘hypertension’, ‘high blood pressure’, ‘medical imaging’, and ‘machine learning’ (see Supplementary material online, *Tables S1–S7*). MEDLINE, EMBASE (via Ovid), Scopus, PubMed, IEEE Xplore, CINAHL, and Web of Science were searched automatically and manually from inception to December 2025. One reviewer (M.A.) conducted the searches without language restrictions. Reference lists of retrieved articles were manually screened to ensure study eligibility.

Eligibility criteria required studies to (i) be diagnostic or prognostic in design, (ii) involve human adults (>18 years), (iii) report on organ structure or function, and (iv) apply AI for identifying, phenotyping, or predicting hypertension and its outcomes. Exclusion criteria included (i) conference abstracts, reviews (narrative/systematic), meta-analyses, editorials, letters, comments, and retracted papers and (ii) research focused on pulmonary hypertension or hypertensive disorders of pregnancy. No restrictions were placed on imaging modality or organ studied.

### Intervention and outcomes

The population included adults diagnosed with elevated blood pressure or hypertension. Interventions involved AI and computational tools, including supervised or unsupervised machine learning, applied to population data for metric extraction, risk prediction, diagnosis, subgroup phenotyping, characterizing the disease, or predicting its adverse outcomes. Tools ranged from simple computational models to more advanced deep learning algorithms. The primary outcome was AI-extracted image features describing structural or functional changes across hypertension stages. Secondary outcomes included AI and computational applications in medical imaging for assessing disease progression, predicting outcomes, and evaluating automated diagnostic performance, either by pre-extracted features (imaging biomarkers) or features extracted directly by AI and computational algorithms.

### Selection of studies

All records identified through database searches were deduplicated using the Systematic Review Accelerator,^15^ followed by manual removal based on titles and abstracts. Three investigators (M.A., P.D.S., and H.R.C.) screened abstracts using Rayyan software.^16^ Full-text eligibility was assessed to apply inclusion and exclusion criteria. Selection conflicts were reviewed by a fourth investigator (W.L.) and resolved through discussion among all reviewers.

### Data extraction

A standardized extraction form was used to collect data from all articles, with extraction performed by a single reviewer (M.A.) and independently verified by two additional reviewers (P.D.S. and H.R.C.) to ensure accuracy and completeness. The extracted data included first author, publication year, country of study conduction, study design, dataset size, age range, female percentage, organ type, imaging modality, hypertension diagnosis, pre-processing steps, type of AI, task of AI, performance metrics, outcome measures, and external validation results. For articles with missing details or ambiguous information, the authors were directly contacted for further clarification.

### Risk of bias assessment

We conducted risk of bias assessment to systematically evaluate the reliability and applicability of the methodological quality and potential of studies in the context of AI-driven and computational medical imaging for hypertension. This allows for weighing the studies based on their influence in the interpretation and synthesis of provided evidence.^17^ The quality of the included studies was assessed based on two tools: the Quality Assessment of Diagnostic Accuracy Studies Artificial Intelligence (QUADAS-AI)^18^ for diagnostic studies and the Prediction model Risk of Bias Assessment Tool (PROBAST)^19^ for prognostic studies. Three reviewers (M.A., P.D.S., and H.R.C.) independently evaluated quality of the articles based on risk of bias, applicability concerns, and overall representation according to the two assessment tools. If there were conflicts between the reviewers, e.g. conflict on bias score, a fourth independent reviewer (W.L.) resolved them after discussion by selecting the best assessment.

The QUADAS-AI criteria included (i) patient selection, (ii) interpretation of index tests, (iii) reference standard, and (iv) flow/timing, while PROBAST assessed (i) inclusion/exclusion of participants, (ii) predictor selection, (iii) outcome standard, and (iv) model performance analysis. Each criterion was assigned a score of −1 (high risk), 0 (unclear risk), or 1 (low risk). For the overall evaluation of bias and applicability, if any criterion was rated as high risk, i.e. −1, the study was classified as high risk overall. If only unclear risk, i.e. 0, was assigned alongside low risk, i.e. 1, the study was categorized as unclear risk overall. A study was considered low risk overall only if all criteria were individually rated as low risk (1).

### Thematic data synthesis

All identified studies were grouped into categories based on their core objective in applying AI and computational algorithms to medical imaging for hypertension research. We examined the primary tasks performed by AI, defining a task as a separate category if addressed by at least three studies to ensure statistical validity. A thematic analysis was then conducted by reviewing each study’s objectives, methods, and findings to identify and group similar AI tasks into thematic categories. This synthesis allowed comparison across studies despite differences in design, imaging modalities, or AI techniques. The final grouping was reached through consensus among three reviewers (M.A., P.D.S., and H.R.C.), with disagreements resolved through discussions with independent reviewers (W.L., G.M., and P.L.). Additional analyses were performed separately for each task category.

### Meta-analysis

We conducted meta-analysis on studies grouped by thematic categories, primarily focusing on (i) differentiating hypertension from normotension and (ii) distinguishing hypertension from hypertrophic cardiomyopathy (HCM). We evaluated these two categories based on the availability of a sufficient number of studies reporting performance and addressing a common objective. The meta-analysis was conducted using hierarchical random-effects models to evaluate sensitivity across studies, while accounting for variability, using contingency data where available.

Sensitivity was first synthesized using a univariate random-effects model, and heterogeneity was assessed using the *I*^2^ statistic from Cochran’s Q test,^20^ with thresholds of low (25–49%), moderate (50–74%), and high (>75%) heterogeneity. Statistical significance was tested using the Cochran’s *Q* test and defined as a *P* value < 0.05 indicating significant heterogeneity. Sensitivity forest plots were generated in R using the ‘Meta’ package (version 8.0-1). For the sensitivity calculations, true positives (TP) were treated as the ‘Events’ and the ‘Total’ was defined as the sum of TP and false negatives (FN).

Furthermore, to jointly synthesize sensitivity and specificity, we applied a bivariate random-effects model (Reitsma model). A summary receiver operating characteristic (SROC) curve^21^ with a 95% confidence interval was generated, and the area under the curve (AUC) was calculated as an overall performance measure. These analyses were performed using the ‘Mada’ package in R. Lastly, funnel plots were examined to evaluate performance symmetry.

## Results

### Search results

**Figure 1:**
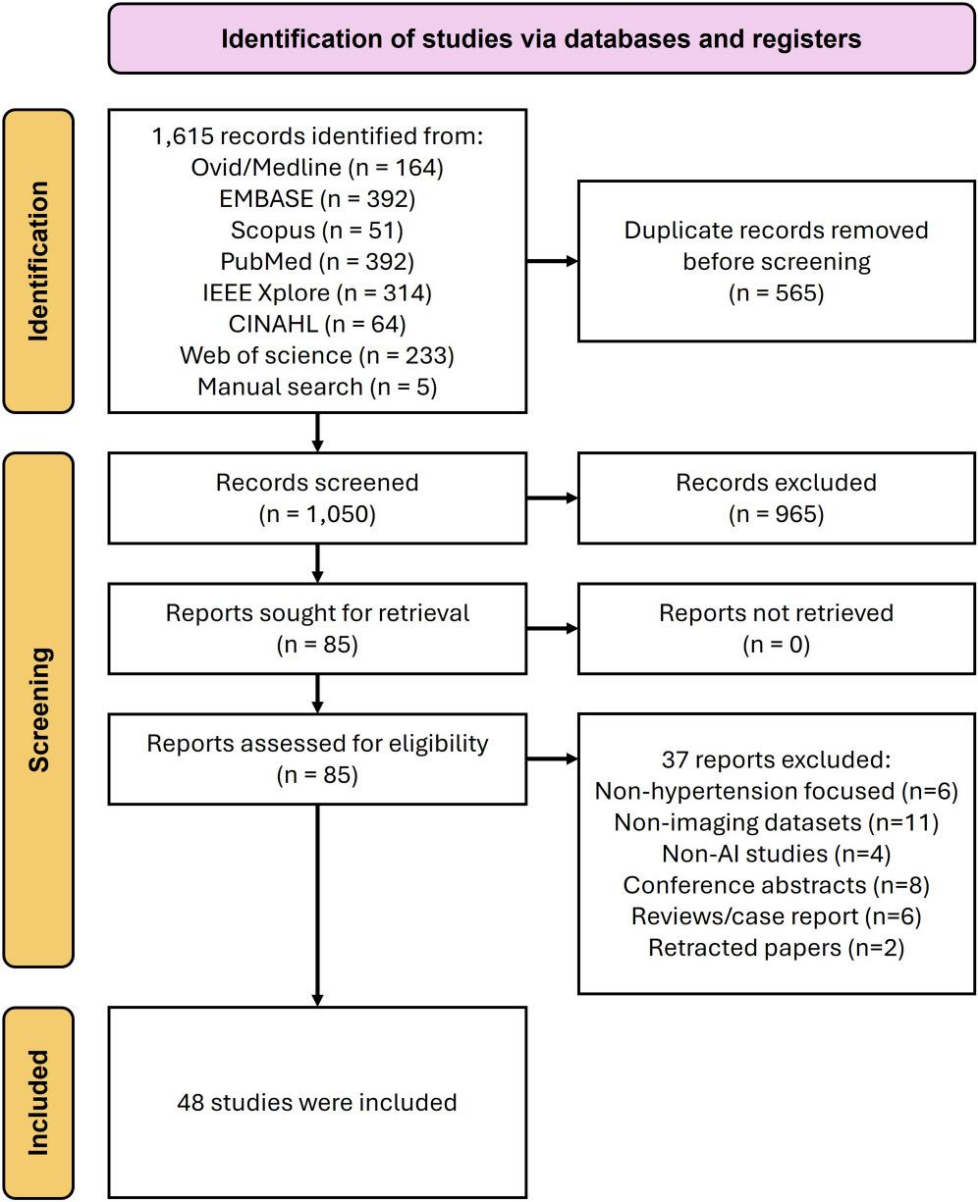
Flow diagram of the study selection process. Out of the 1615 records identified by the search strategy and manual searching, 48 studies were eligible and included for analysis.

The initial database search identified 1615 records. After removing duplicates, 1050 records were screened by title and abstract, resulting in the exclusion of 965 records. Full texts were retrieved for 85 articles, of which 37 were excluded after detailed eligibility assessment. Finally, 48 articles were included for further analysis (*Figure 1*).

### General characteristics of included studies

A detailed summary of the characteristics of the 48 included studies is presented in Supplementary material online, *Figure S1*. Most of the selected studies were conducted after 2017 (*n* = 41; 85.42%). The majority of corresponding/first authors were from China (*n* = 11; 22.92%) and the USA (*n* = 8; 16.67%). Of the included studies, 25 studied the heart (52.08%), followed by 12 focusing on the retina (25.00%) and 6 on the brain (12.50%). The imaging modalities varied, with echocardiography being the most common (*n* = 16; 33.33%), followed by retinal imaging (*n* = 12; 25.00%).

In the risk of bias assessment (see Supplementary material online, *Figure S2*), 25 studies (52.08%) were classified as having low risk, 3 studies (6.25%) as unclear risk, and 20 studies (41.67%) as high risk of bias. Similarly, applicability assessment showed that 25 studies (52.08%) had low risk. The breakdown of these results per-study is provided in Supplementary material online, *Table S8*.

The included studies utilized various AI models, mainly for classification tasks (see Supplementary material online, *Figure S3*). Most studies used supervised learning (*n* = 40; 83.33%) for model training. Studies were evenly divided between traditional machine learning (*n* = 24; 50.00%) or deep learning methods (*n* = 24, 50.00%). Among traditional approaches, ensemble learning (*n* = 9) and support vector machines (*n* = 9) were most common, whereas deep learning studies primarily applied convolutional neural networks (CNN) (*n* = 8).

### Thematic analysis

We categorized studies into five major tasks (*Table 1*) as follows: AI Task 1) identifying hypertension or its stages—10 studies (21%); AI Task 2) differentiating hypertension from other diseases/conditions—19 studies (40%); AI Task 3) characterizing target end-organ changes associated with hypertension—11 studies (23%); AI Task 4) identifying subgroups and phenotypes within hypertensive cohorts—4 studies (8%); and AI Task 5) assessing the risk of future hypertension-related outcomes—3 studies (6%). A single study focusing on estimating blood pressure levels^69^ was categorized under other applications. A detailed breakdown of these studies is provided in Supplementary material online, *Tables S9* and *S10* by organ and by AI techniques, respectively.

**Table 1:** Summary of studies based on major thematic tasks to characterize hypertension

Study	Year	Target population	Type of modality	Outcome of interest
AI Task 1 Identifying hypertension or its stages				
Retinal microvasculature				
Morales *et al*.^22^	2014	Normal HR with at least one sign of pathological arteriovenous crossing	Retinal imaging	Discriminate between healthy fundus and hypertensive
Lo *et al*.^23^	2021	Normal HR with Mitchell-Wong grading system standards		Identify mild hypertension using retinal images
Brain				
Yu *et al*.^24^	2019	Hypertensives with four grades based on ACC 2017 guidelines	Brain MR	Discriminate between different hypertension grades by brain structural changes
Kandil *et al*.^25^	2019	Normal Pre-hypertensive with SBP: 120–140 and DBP: 80–90		Identify hypertension based on cerebral vascular changes
Kandil *et al*.^26^	2020	Normal Hypertensive with SBP: >130 and DBP: >80		Identify hypertension by tracking the cerebral vascular alterations
Kandil *et al*.^27^	2020	Normal Hypertensive with SBP: >130 and DBP: >80		Identify hypertension using brain vascular features and SBP/DBP groups
Peng *et al*.^28^	2020	Hypertensives with four grades based on ACC 2017 guidelines		Discriminate between different hypertension grades using brain structural changes
Heart				
Cetin *et al*.^29^	2019	Normal Hypertensive with no cardiovascular diseases	Cardiac MR	Identify hypertension using image radiomics
Wang *et al*.^30^	2022	Hypertensives with four grades based on ESC/ESH 2018 guidelines	Cardiac CT	Identify hypertension using aortic diameter measurements
Tongue				
Karki *et al*.^31^	2020	Normal Hypertensive with liver, kidney, and spleen problems	Tongue images	Identify hypertension using tongue images
AI Task 2 Differentiating hypertension from other diseases				
Retinal microvasculature				
Rose *et al*.^32^	2023	HR patients Patients with heart attack	Retinal imaging	Discriminate between hypertensive retinopathy and heart attack
Koduri *et al*.^33^	2024	HR patients		Discriminate between hypertensive retinopathy and diabetic retinopathy
Heart				
Azhari *et al.*^34^	1991	HT with LVH and/or WMA Myocardial infarction—LV aneurysms	Cardiac CT	Discriminate hypertension from different pathological conditions
Neisius *et al*.^35^	2019	HT with increased LVWT (>12 mm) Hypertrophic cardiomyopathy	Cardiac MR	Discriminate between hypertensive heart disease and hypertrophic cardiomyopathy
Cetin *et al*.^36^	2020	Normal Hypertensives defined by self-reporting conditions	Cardiac MR	Identify hypertension from changes in cardiac structure and tissue texture
Vidal-Sospedra *et al*.^37^	2020	Hypertensive heart disease Hypertrophic cardiomyopathy Amyloidosis	Cardiac MR	Differentiate between HCM, HIP, and AM using image texture analysis
Yu *et al*.^38^	2020	Hypertensive heart with LVH Hypertrophic cardiomyopathy (LVWT ≥15 mm) Uraemic cardiomyopathy with chronic ESRD	Echocardiography	Discriminate between hypertensive heart disease and other heart diseases
Sabovčik *et al*.^39^	2021	Hypertensive with SBP: >140, DBP: >90 and using antihypertensive drugs Patients with LVDD and LVH	Echocardiography	Discriminate between LVH and LVDD based echocardiographic ground truth information
Shi *et al*.^40^	2021	HT with increased LVWT (>12 mm) Hypertrophic cardiomyopathy (LVWT ≥15 mm)	Cardiac MR	Discriminate between HCM and HHD using image texture analysis
Forghani *et al*.^41^	2021	Hypertensive heart diagnoses in hospital	Echocardiography	Discriminate between HCM and HHD using echocardiography and ECG
Barbieri *et al*.^42^	2022	Arterial hypertension with diabetes or other comorbidities	Echocardiography	Determine changes in left atrial and left ventricular in response to hypertension
Hwang *et al*.^43^	2022	HT with LVH and LVWT >12 mm Hypertrophic cardiomyopathy (LVWT ≥15 mm) AM	Echocardiography	Discriminate between hypertensive heart disease and other heart diseases
Xu *et al*.^44^	2022	Hypertensive heart with history of systemic hypertension or aortic valve stenosis	Echocardiography	Discriminate between hypertensive heart disease and normotensives
Zhang *et al*.^45^	2023	HT with history of hypertension Hypertrophic cardiomyopathy (LVWT ≥15 mm) Uraemic cardiomyopathy with eGFR <15 mL/min/1.73 m^2^ AM	Echocardiography	Discriminate between hypertensive heart disease and other heart diseases
Diao *et al*.^46^	2023	HT with increased LVWT (>12 mm) Hypertrophic cardiomyopathy (LVWT ≥15 mm) AM	Cardiac MR	Discriminate between hypertensive heart disease and other heart diseases
Wang *et al*.^47^	2024	HT with increased LVWT (>12 mm) with no other cardiac diseases Hypertrophic cardiomyopathy (LVWT ≥15 mm)	Cardiac MR	Discriminate between HHD and HCM using cardiac images
Wang *et al*.^48^	2024	HT with increased LVWT (>12 mm) with no other cardiac diseases History of prolonged uncontrolled arterial hypertension	Cardiac MR	Discriminate between HHD and 10 CVDs using cardiac images
Moon *et al*.^49^	2025	HT subjects had hypertension with LVH confirmed by echocardiography	Echocardiography	Discriminate between HHD and HCM using echocardiography features
Vasculature				
Recenti *et al*.^50^	2021	Normal Hypertensive and diabetic	Femoral CT	Identify hypertension from muscle radiodensitometry in CT
AI Task 3 Characterizing target end-organ changes				
Retinal microvasculature				
Kaupp *et al*.^51^	1994	Hypertensive with SBP: >130 and DBP: >80 and minimal retinal vascular alterations.	Retinal imaging	Measurement of morphological properties of retinal vessels
Khitran *et al*.^52^	2014	Normal A/V ratio HR with 0.1–0.5 A/V ratio		Measurement of morphological properties of retinal vessels
Ahmad *et al*.^53^	2018	Normal A/V ratio HR with abnormal A/V ratio		Classification of arteries and veins
Kiruthika *et al*.^54^	2019	Normal A/V ratio HR with abnormal A/V ratio		Classification of arteries and veins
Dai *et al*.^55^	2020	Hypertensive with SBP: >140 and DBP: >90 with history of hypertension or medication with no comorbidities		Identify hypertension using retinal images
Bhimavarapu *et al*.^56^	2024	HR patients with no other retinal diseases HR of stages mild, moderate, severe, and malignant		Discriminate between hypertensive retinopathy at different stages
Triwijoyo *et al*.^57^	2025	Diabetic retinopathy labelled into nine classes HR based AVR		Discriminate between hypertensive retinopathy at different stages
Heart				
Raghavendra *et al*.^58^	2022	Normal HT without renal failure, coronary, congenital, or valvular disease	Echocardiography	Predict hypertension using structural alteration
Alsharqi *et al*.^59^	2023	Hypertensive with SBP: >160		Generate clinically meaningful scores and phenotypes of cardiac remodelling
Vasculature				
Pessana *et al*.^60^	2010	Hypertensives without any medications	Carotid ultrasound	Assessment of instantaneous arterial diameter and WAVP in hypertension
Multi-organ, *i.e.* heart, brain, liver, kidney, and vasculature				
Alkhodari *et al*.^61^	2023	Hypertensive with SBP: >160 and DBP: >100 with no comorbidities	Cardiac MR, brain MR, and carotid ultrasound	Generate a unified measure to identify multi-organ phenotypes and trajectories
AI Task 4 Identifying phenotypes and hypertension subgroups				
Brain				
Yang *et al*.^62^	2024	Hypertensive with SBP: >130 and DBP: >80 with history of hypertension or medication	Brain MR	Discovering hypertension subgroups using imaging and genetic data
Heart				
Katz *et al*.^63^	2017	Hypertensive with SBP: >140 and DBP: >90 with no comorbidities	Echocardiography	Identify hypertensive subgroups with varying myocardial substrates
Loncaric *et al*.^64^	2021	Hypertensive with history of antihypertensive treatment and no comorbidities		Identify functional phenotypes in velocity profiles of hypertension
Rauseo *et al*.^65^	2026	HT defined using ICD codes, self-reported disease, doctor-diagnosed conditions, and the use of antihypertensive medications up until the time of the first imaging visit. Blood pressure values alone were not used to define HT	Cardiac MR	Identify hypertensive subgroups with varying myocardial substrates
AI Task 5 Defining risk of future hypertension-related events				
Retinal microvasculature				
Squirrell *et al*.^66^	2024	No prior ASCVD	Retinal imaging	Predict an individual SBP from retinal image and correlate it with future ASCVD events
Heart				
Giovanna *et al*.^67^	2002	Hypertensive with SBP: >140 and DBP: >90	Echocardiography	Cardiovascular risk stratification using microalbuminuria and echocardiography
Viazzi *et al*.^68^	2006	Hypertensive with history of antihypertensive treatment	Echocardiography	Cardiovascular risk stratification using creatinine and echocardiography
Other additional Applications				
Vasculature				
Jana *et al*.^69^	2020	Normal and low BP Elevated and HT with stages 1 or 2	Doppler ultrasound	Identify blood pressure levels using cuffless estimation and Doppler ultrasound

A total of 48 studies were included in this systematic review.

SBP, systolic blood pressure; DBP, diastolic blood pressure; HR, hypertensive retinopathy; MR, magnetic resonance; CT, computed tomography; ACC, American College of Cardiology; ESC/ESH, European Society of Cardiology and European Society of Hypertension; HT, hypertension; CVD, cardiovascular disease; LV, left ventricle; HCM, hypertrophic cardiomyopathy; HIP, hypertensive cardiomyopathy; AM, amyloidosis; HHD, hypertensive heart disease; LVH, left ventricular hypertrophy; LVDD, left ventricular diastolic dysfunction; WMA, wall motion abnormality; LVWT, left ventricular wall thickness; ESRD, end-stage renal disease; eGFR, estimated glomerular filtration rate; A/V ratio, arteriovenous ratio; HICH, hypertensive intracerebral haemorrhage; mRS, modified Rankin scale; ASCVD, atherosclerotic cardiovascular disease.

#### AI task 1: identifying hypertension or its stages

**Figure 2:**
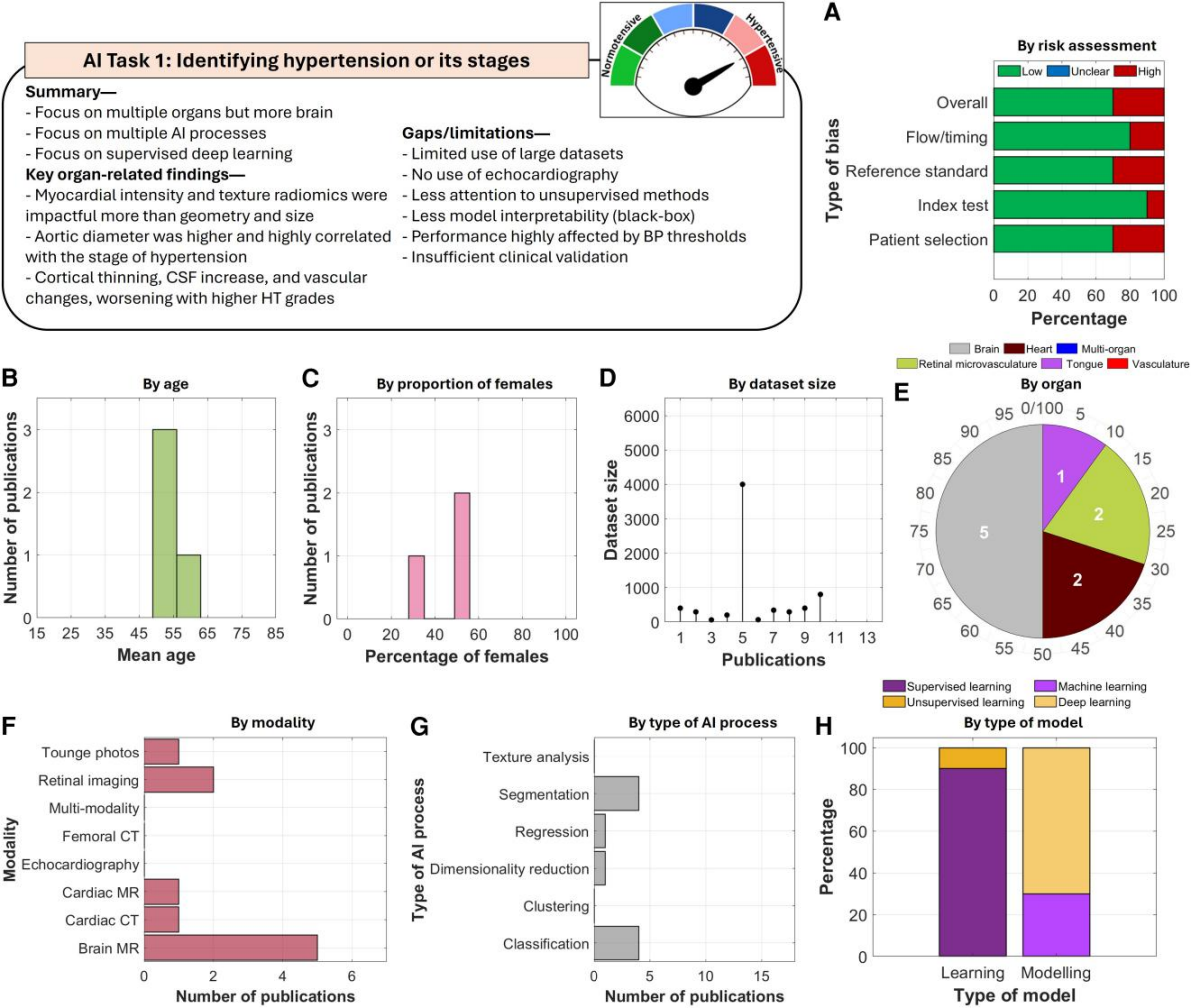
Thematic analysis of the included studies in AI task 1. A total of 10 studies were included in this task. Analysis of (*A***)** risk of bias, (*B***)** by age, (*C***)** proportion of females, (*D***)** size of datasets, (*E***)** organs being imaged, (*F***)** imaging modalities, (*G***)** tasks performed by the AI models, and (*H***)** types of learning and modelling.

In this task, studies examined multiple organs, with a primary focus on the brain (*Figure 2*). Heart studies using cardiac MR identified intensity- and texture-based radiomics as impactful metrics for identifying hypertension,^29^ outperforming geometric parameters of the myocardium. Furthermore, aortic diameter was higher in hypertensive cases and strongly correlated with the severity of hypertension^30^ using cardiac CT. In brain imaging, cortical thinning, cerebrospinal fluid increase, and vascular alterations worsening with higher hypertension grades.^24^ Moreover, progressive structural changes, mainly reductions in volume, were varied by hypertension severity.^28^ A single study investigated the use of tongue imaging, reporting a strong association between vascular features in the tongue and concurrent liver and kidney abnormalities.^31^

**Figure 3:**
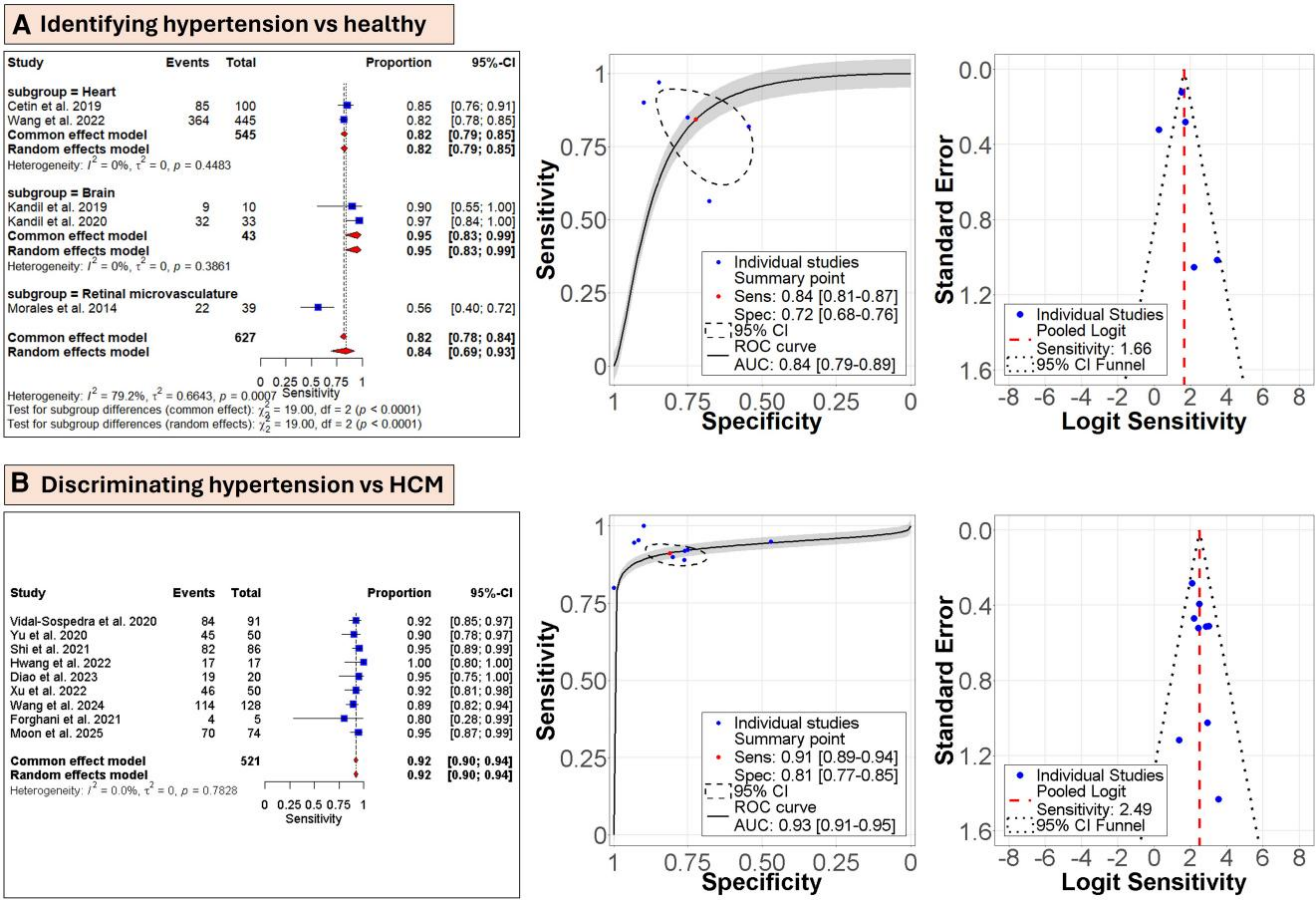
Meta-analysis and summary receiver operating characteristics (SROC) curves of the included studies with their corresponding funnel plots. (*A*) Identifying hypertension vs. healthy conditions in AI Task 1 (5 studies). The analysis was also based on organ subgrouping. (*B*) Discriminating hypertension from hypertrophic cardiomyopathy (HCM) in AI Task 2 (9 studies). Only studies that reported performance were included. For the sensitivity calculations, true positives (TP) were treated as the ‘Events’ and the ‘Total’ was defined as the sum of TP and false negatives (FN). Each SROC was provided with 95% confidence interval, summary point for sensitivity and specificity, and overall area under the ROC curve (AUC). Funnel plots were used to check symmetry between studies in their reported performance.

Most studies employed supervised learning-based models, with the majority relying on deep learning. Overall, they demonstrated a low risk of bias. Models were primarily used for classification, often preceded by segmentation. However, several gaps remain in this body of research. Datasets were relatively small, unsupervised learning approaches were rarely explored, performance was highly influenced by blood pressure thresholds, and the models were purely predictive with limited interpretability.

Meta-analysis was conducted on studies that focused on identifying hypertensive patients from normotensives (*Figure 3A* and Supplementary material online, *Table S11*). Included studies used data on the heart, brain, and retinal microvasculature. The heart subgroup had a sensitivity of 0.82 [0.79–0.85], the brain showed the highest values at 0.95 [0.83–0.99], and the retinal microvasculature had a single study with low sensitivity of 0.56 [0.40–0.72]. The overall sensitivity was 0.84 [0.69–0.93], with overall heterogeneity of 79.2% and no heterogeneity in per-organ analysis. The SROC curve revealed an AUC of 0.84 [0.79–0.89], while the funnel plot demonstrated symmetry with logit sensitivity of 1.66.

#### AI task 2: differentiating hypertension from other diseases

**Figure 4:**
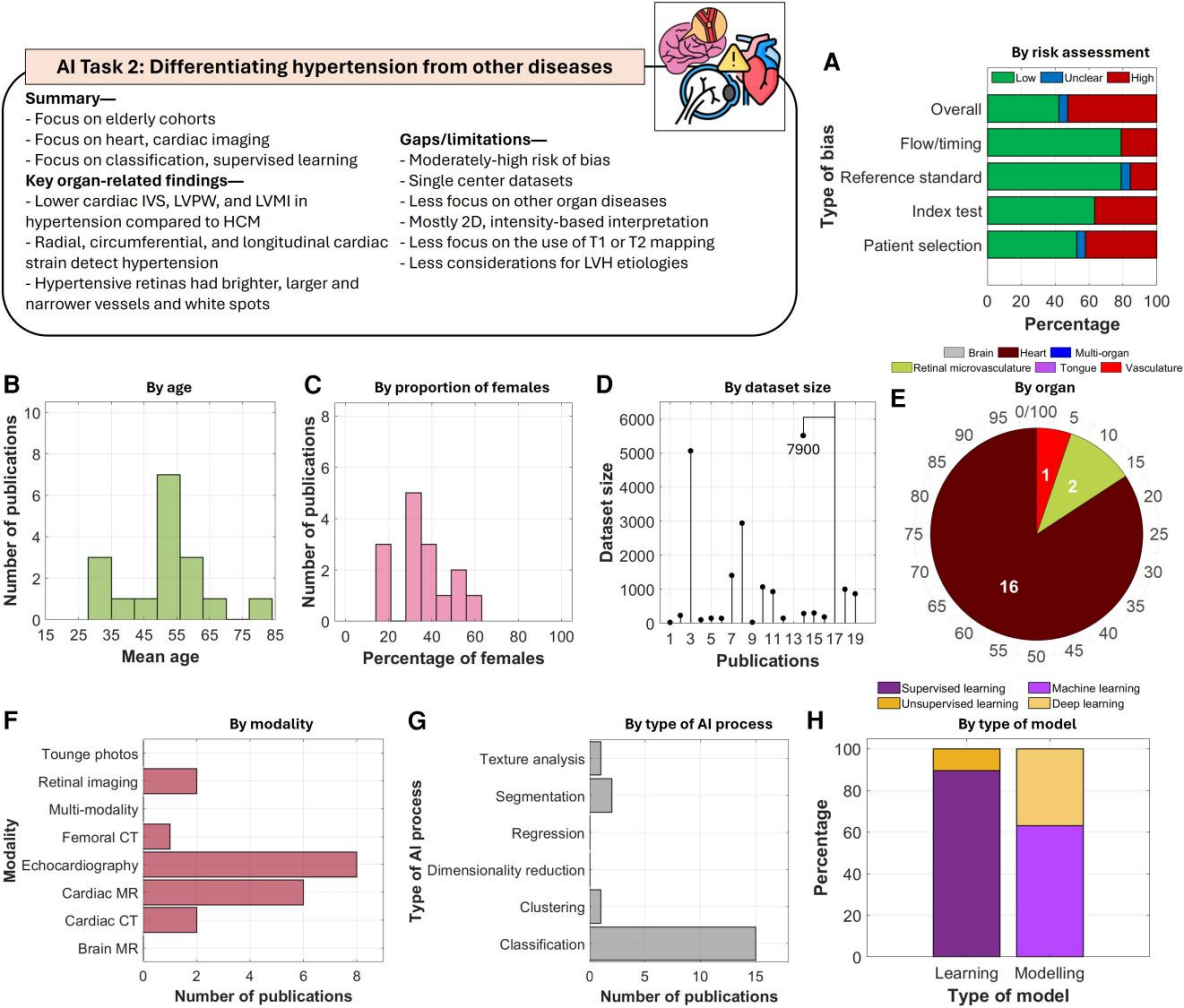
Thematic analysis of the included studies in AI task 2. A total of 19 studies were included in this task. Analysis of (*A***)** risk of bias, (*B***)** by age, (*C***)** proportion of females, (*D***)** size of datasets, (*E***)** organs being imaged, (*F***)** imaging modalities, (*G***)** tasks performed by the AI models, and (*H***)** types of learning and modelling.

Studies mainly addressed distinguishing hypertensive disease from other cardiovascular conditions, particularly HCM, using cardiac imaging (*Figure 4*). Most focused on the heart using modalities such as cardiac MR, echocardiography, and cardiac CT. Key hypertension markers included reduced IVS thickness, LVPW thickness, and LV mass index (LVMI) compared with HCM.^45,46^ Moreover, radial, circumferential, and longitudinal strain parameters,^40^ late gadolinium enhancement on SAX,^48^ and time/frequency domain contractility features^44^ were effective in distinguishing hypertension. In retinal imaging, severe hypertensive retinopathy (HR) was characterized by brighter, wider vessels with white spots.^32^ Additionally, narrower arterioles helped distinguish HR from diabetic retinopathy.^33^

In this task, supervised classification approaches were more common. Models ranged from traditional machine learning to advanced deep learning architectures, including attention-based transformers.^48^ The overall risk of bias remained moderately high at 52.6%. Several limitations and gaps in current literature should be noted. First, most studies focused solely on the heart. Moreover, models relied on two-dimensional (2D) imaging with intensity-based interpretations, whereas use of three-dimensional (3D) was lacking, highlighting an important direction for future research to explore 3D imaging approaches. Finally, there was limited evaluation of specialized sequences such as T1/T2 mapping or consideration of other LVH aetiologies.

Meta-analysis here involved studies that distinguished hypertension from HCM (*Figure 3B* and Supplementary material online, *Table S11*). Nine studies were included in the analysis, demonstrating an overall sensitivity of 0.92 [0.90–0.94]. No heterogeneity was found between the studies. The AUC was at 0.93 [0.91–0.95] with funnel plot symmetry around logit sensitivity of 2.49.

#### AI task 3: characterizing target end-organ changes

**Figure 5:**
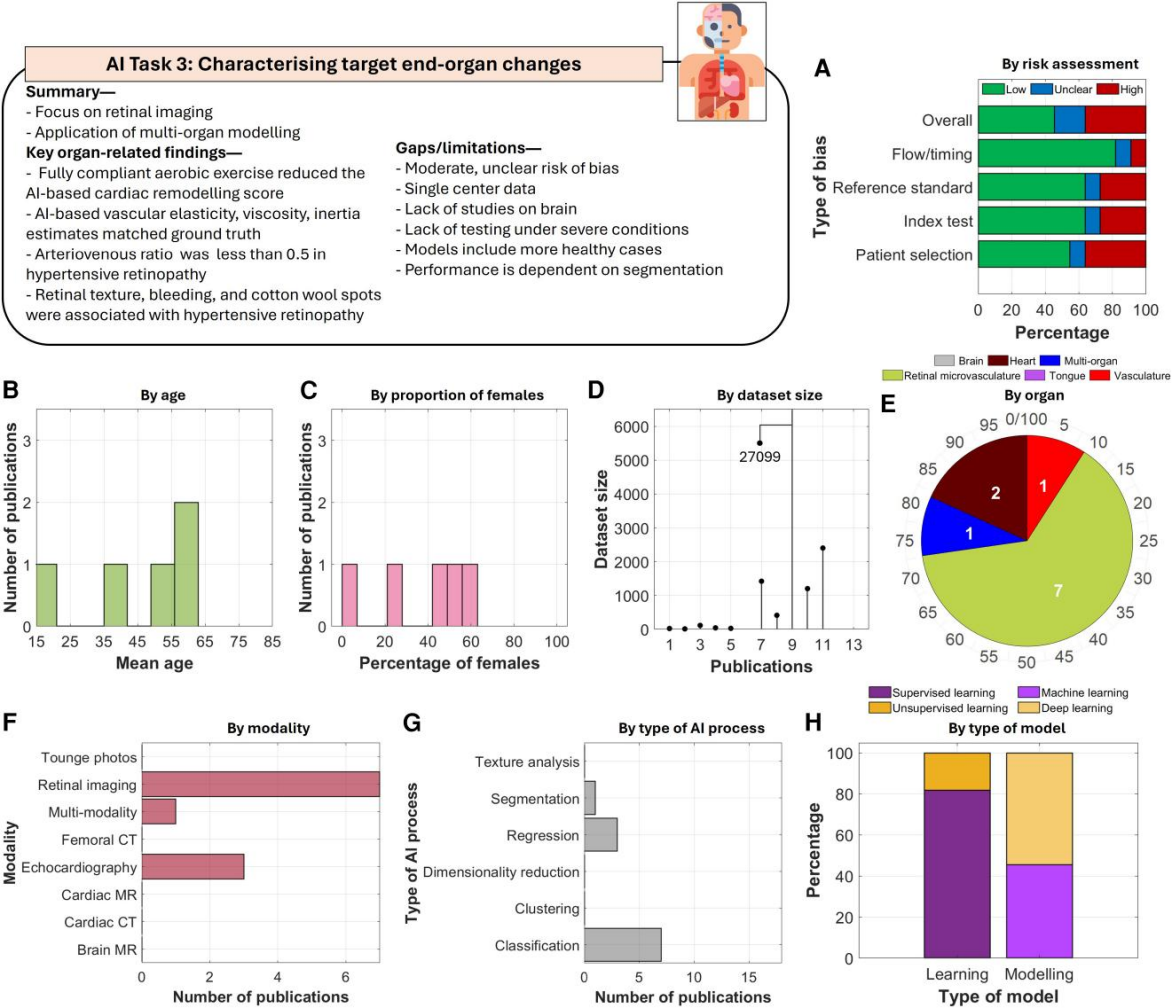
Thematic analysis of the included studies in AI task 3. A total of 11 studies were included in this task. Analysis of (*A*) risk of bias, (*B*) by age, (*C*) proportion of females, (*D*) size of datasets, (*E*) organs being imaged, (*F*) imaging modalities, (*G*) tasks performed by the AI models, (*H*) types of learning and modelling.

The primary goal of this task was to detect organ alterations using AI and validate its effectiveness in identifying evidence of end organ changes related to hypertension (*Figure 5*). Retinal imaging was the most common imaging studied, with a limited number on cardiac and vascular imaging. Within cardiac imaging, geometrical properties derived from the SHELet transform were found key markers of hypertension.^58^ In retinal imaging, alterations in arteriovenous ratio below 0.5,^52^ approximately 25% greater arterial tortuosity and significantly reduced arterial diameter were identified.^51^ Moreover, changes near the optic nerve and images with thin vessels,^56^ alongside changes in retina texture, blood vessels, hard exudates, bleeding, and cotton wool spots^57^ were identifiable. A single study integrated multi-organ information using multi-modality imaging in an AI model,^61^ identifying unique hypertension associations with white matter hyperintensities (WMH), grey matter volume, and intima-media thickness.

The overall risk of bias in this task was moderate, with 18% of studies classified as having unclear risk. Studies under this task had several limitations. None examined the brain, models included a limited number of subjects with severe hypertension or complex disease scenarios, and predictive performance relied heavily on the quality of segmentation and image analysis.

#### AI task 4: identifying phenotypes and hypertension subgroups

**Figure 6:**
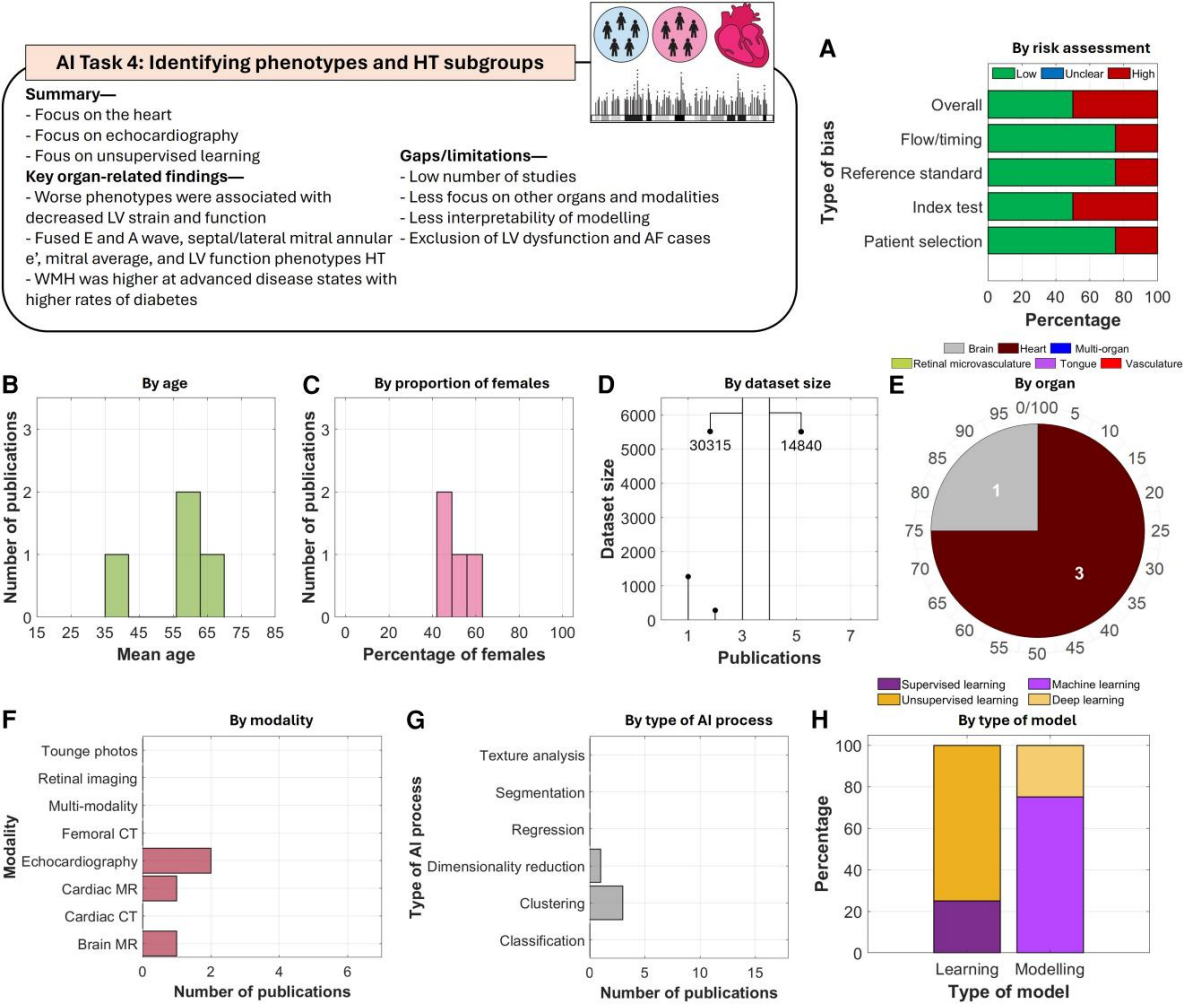
Thematic analysis of the included studies in AI task 4. A total of four studies were included in this task. Analysis of (*A***)** risk of bias, (*B***)** by age, (*C***)** proportion of females, (*D***)** size of datasets, (*E***)** organs being imaged, (*F***)** imaging modalities, (*G***)** tasks performed by the AI models, and (*H***)** types of learning and modelling.

Studies under this task focused mostly on the heart using echocardiography (*Figure 6*). AI-based cardiac analysis identified distinct hypertension-related phenotypes, including one associated with worse outcomes and abnormalities in LV mass, LV end-diastolic dimension (LVED), LV stroke volume (LVS), LV wall thickness (LVWT), and reduced longitudinal strain.^63^ Unique phenotypes were also characterized by significant differences in fused E and A waves, septal/lateral mitral annular e’, mitral average, and LV function.^64^ Moreover, hypertensive cardiac alterations were found to form three clusters/phenotypes as: minimal cardiac remodelling, moderate metabolic syndrome changes, and severe cardiac alterations with atherosclerosis.^65^ A single study identified five distinct subtypes using brain MRI,^62^ with two advanced subtypes showing higher WMH and increased rates of diabetes, while no differences were observed in hyperlipidaemia.

In contrast to other tasks, as expected, this task more prominently employed unsupervised machine learning. Identified gaps included a limited number of studies, with most not exploring phenotyping based on organ characteristics beyond the heart. Only a single study applied deep learning-based analysis^62^ that culminated in clustering and phenotype discovery, leaving room to explore more advanced techniques. Additionally, models often excluded patients with severe disease, e.g. LV dysfunction and atrial fibrillation. Finally, given the reliance on machine learning models, interpretability was often limited, with explanations mostly derived from clustering analysis of identified phenotypes.

#### AI task 5: defining risk of future hypertension-related events

**Figure 7:**
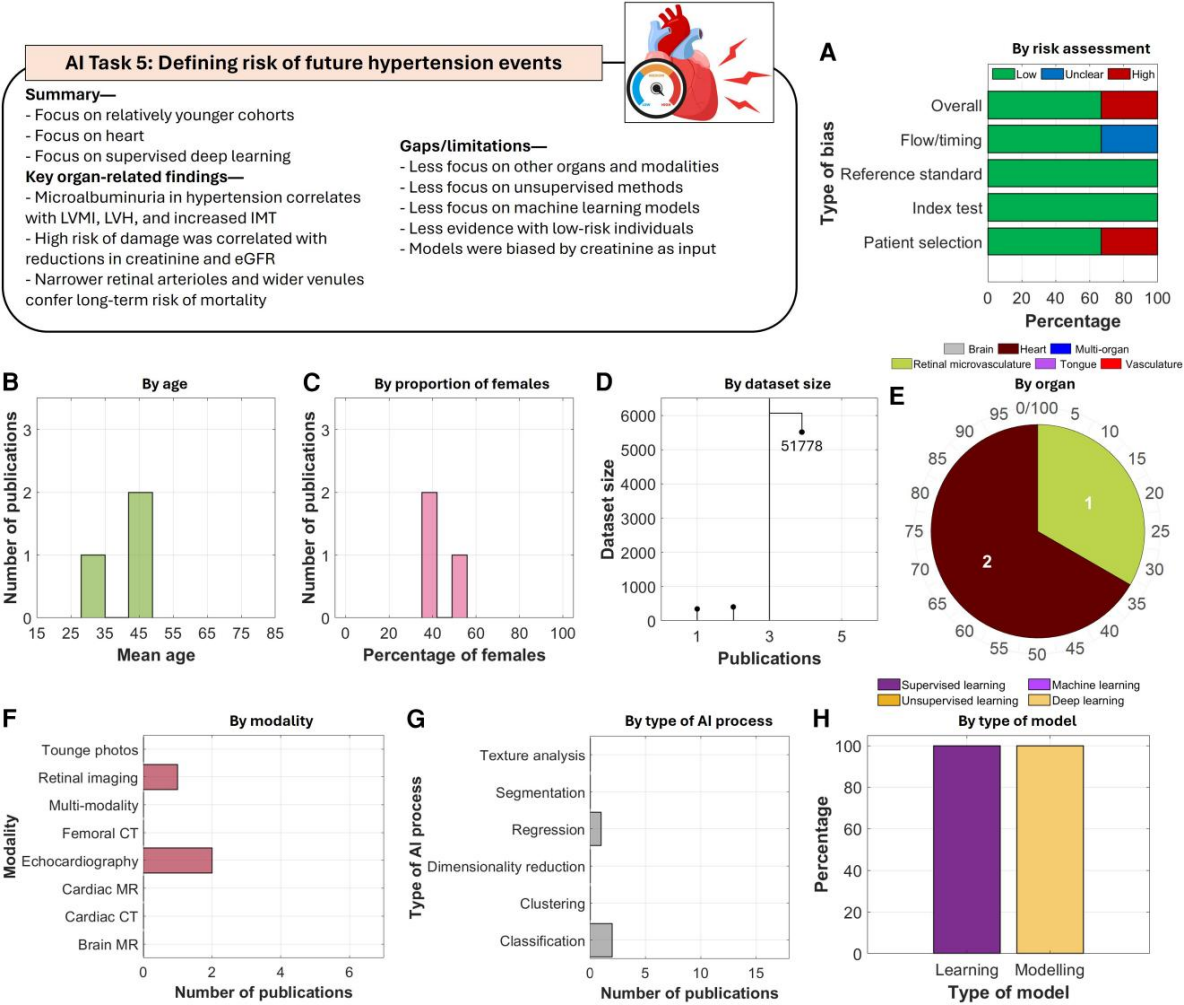
Thematic analysis of the included studies in AI task 5. A total of three studies were included in this task. Analysis of (*A*) risk of bias, (*B*) by age, (*C*) proportion of females, (*D*) size of datasets, (*E*) organs being imaged, (*F*) imaging modalities, (*G*) tasks performed by the AI models, and (*H*) types of learning and modelling.

This task included studies focusing on the heart (using echocardiography) and the retina, to predict future risk and adverse outcomes (*Figure 7*). Heart studies predominantly focused on associations with risk assessed by other surrogate measures such as renal function, for example, reduced creatinine clearance and estimated glomerular filtration rate^68^ or microalbuminuria.^67^ In retinal imaging study, narrower retinal arterioles and wider venules conferred long-term risk of mortality.^66^

This task was relatively underexplored. All studies employed supervised deep learning approaches. Several gaps remain in this task. There was limited exploration of other organs, especially the brain, for predicting future adverse outcomes. No studies employed processes beyond classification, such as segmentation or dimensionality reduction. Moreover, unsupervised machine learning algorithms were not utilized. Finally, the models provided limited evidence for predicting future risk in low-risk individuals, as they often included patients with severe hypertension.

## Discussion

In this systematic review, 48 studies were identified that examined the integration of AI and computational algorithms with imaging to enhance hypertension identification, phenotyping, and outcome prediction. The emergence of several thematic areas across these studies underscores the broad applicability of AI and computational algorithms in this context, ranging from hypertension diagnosis and risk stratification to enabling more personalized characterization approaches that move beyond conventional blood pressure-based classifications.

Tasks 1 and 2 explored capability of AI-based assessment of organ changes for detection of hypertension-specific disease. Imaging changes may provide comparatively more stable biomarkers of hypertensive disease state than blood pressure readings.^69^ Our findings demonstrate capability of AI and computational imaging to guide clinical diagnosis, particularly in distinguishing hypertensive end-organ phenotypes from other closely related cardiovascular diseases such as HCM. These diseases often present with overlapping features, posing a diagnostic challenge for clinicians. AI models trained on imaging data also demonstrated strong performance in identifying hypertension-specific organ changes, as outlined in AI Task 3, either by directly learning from the organ images^55^ or by utilizing features pre-extracted from those images.^51^ Therefore, these findings support the potential for translation of AI and computational models into clinical applications to assist with the detection of earlier manifestations of organ pathology. Interestingly, there was limited research characterizing cerebral alterations with AI despite the well-known cerebral changes, including increased white matter hyperintensities and altered cerebral perfusion^62^ linked with hypertension. Further work in this area could significantly enhance AI-driven organ-based models for hypertension detection and management.

A natural next step is the integration of AI into clinical risk stratification frameworks. Existing risk scoring tools, such as QRISK3,^70^ primarily rely on blood pressure, demographics, and laboratory data to estimate future risk of cardiovascular events. However, these scoring systems do not directly take into account severity of target organ damage, which likely contributes significantly to individual risk profiles. Rather than adopting a one-size-fits-all approach, precise phenotyping enables the identification of hypertension subgroups based on organ-specific alterations, paving the way for more personalized and effective management strategies. Current national guidelines for hypertension management, e.g. NICE,^71^ recommend control with blood pressure targets determined by underlying comorbidities, age and estimated cardiovascular risk. Whilst practical, such approaches do not directly account for underlying disease phenotypes. It is plausible that more nuanced approaches to management could be developed with improved risk prediction upon understanding organ-specific phenotypes. To this end, AI methods such as clustering and dimensionality reduction, e.g. *k*-means clustering and principal component analysis, can provide deeper insights into complex datasets, as these approaches are widely regarded as foundational machine learning techniques for uncovering hidden patterns and structures in data.

From a translational perspective, for AI-based and computational imaging tools to be integrated into routine clinical workflows, several challenges need to be addressed.^72^ The use of widely available imaging modalities, such as echocardiography, as opposed to more specialist imaging investigations, may be required to broaden access to the tools. Automation to reduce inter-observer variability and clinician workload will also be important to standardize assessments. However, when implemented effectively, automated analysis of routinely acquired images in outpatient hypertension clinics and primary care settings, by appropriately experienced practitioners, could support early screening for hypertensive end-organ damage and facilitate the identification of high-risk individuals for further evaluation. Future research that prioritizes the development of clinically applicable, simple, yet effective algorithms will enable scalable implementation and enhance adoption in real-world practice.

No studies sought to specify the duration of exposure to hypertension, likely because it is challenging to establish a gold standard measure of hypertension exposure for individuals within the training datasets. This reflects a lack of longitudinal datasets and difficulties with determining onset of early-stage hypertension when it tends to be asymptomatic. Accordingly, none of the studies attempted to describe the changes in organ features occurring over time with repeated imaging. This omission is significant, as individuals with substantial end-organ changes despite a relatively short duration of hypertension may represent an accelerated phenotype,^73^ highlighting the need for further investigation into the temporal progression of hypertensive damage. The knowledge of how these organ alterations occur with time in different individuals could enable earlier risk stratification, potentially preventing progression of hypertension-related long-term outcomes and mortality.

### Study limitations

Our review has limitations. First, despite the overall number of studies included in this systematic review, the number of studies within each subcategory, i.e. task-based or organ-based within tasks, was relatively low, primarily due to the limited application of AI and medical imaging in hypertension care. Second, although the review aimed to cover all organs across various imaging modalities, the current literature has yet to comprehensively explore AI-assisted imaging of certain organs such as kidneys, abdomen, or lungs. Third, most included studies were retrospective or cross-sectional, limiting the ability to assess longitudinal disease progression and AI’s role in long-term hypertension management. Last, most of the included studies suffered from lack of external validation, which increased bias towards their models’ training strategies.

### Future directions

Future research should take into consideration the following aspects. First, studies should investigate the longitudinal progression of hypertension-related organ damage, including using repeated imaging, to better characterize temporal patterns and disease trajectories. Second, although single-organ analyses have revealed important insights, hypertension is a systemic disease that affects multiple organs simultaneously; therefore, future work could focus on developing AI models that integrate multi-organ information. Last, given the ability of AI to uncover previously unrecognized features associated with hypertension, there is a potential that future research could identify novel disease patterns, amenable to personalized lifestyle or pharmacological interventions, that significantly impact individual clinical outcomes.

## Conclusions

This systematic review highlights a nascent potential for AI-driven computational imaging techniques in the early detection, risk stratification, disease phenotyping, and personalized characterization of hypertension, which goes beyond traditional diagnostic approaches. There is a long history of hypertension research, using traditional imaging methods and analysis, that has demonstrated hypertension can impact organ structure and function. The limited body of research applying AI and computational techniques to further develop this understanding is therefore surprising and suggests the potential use of AI for personalized hypertension detection and management lags behind other clinical specialities. This review has identified opportunities for future research to explore the impact of hypertension across multiple body organs. In addition, research could be directed towards identifying different subtypes of hypertensive disease that may benefit from targeted management or drug development.

## Supplementary Material

ztag063_Supplementary_Data

## Data Availability

No new data were generated or analysed in support of this research.
