# Supplementary material for: Applications of artificial intelligence and computational approaches to imaging for hypertension identification, phenotyping, and outcome prediction: a systematic review
Source: Eur Heart J Digit Health. 2026 Apr 20;7(4):ztag063. doi: 10.1093/ehjdh/ztag063 (PMC13175042; doi:10.1093/ehjdh/ztag063)
Supplement: ztag063_Supplementary_Data [file ztag063_supplementary_data.zip › Supplemental_figures_FINAL.docx]

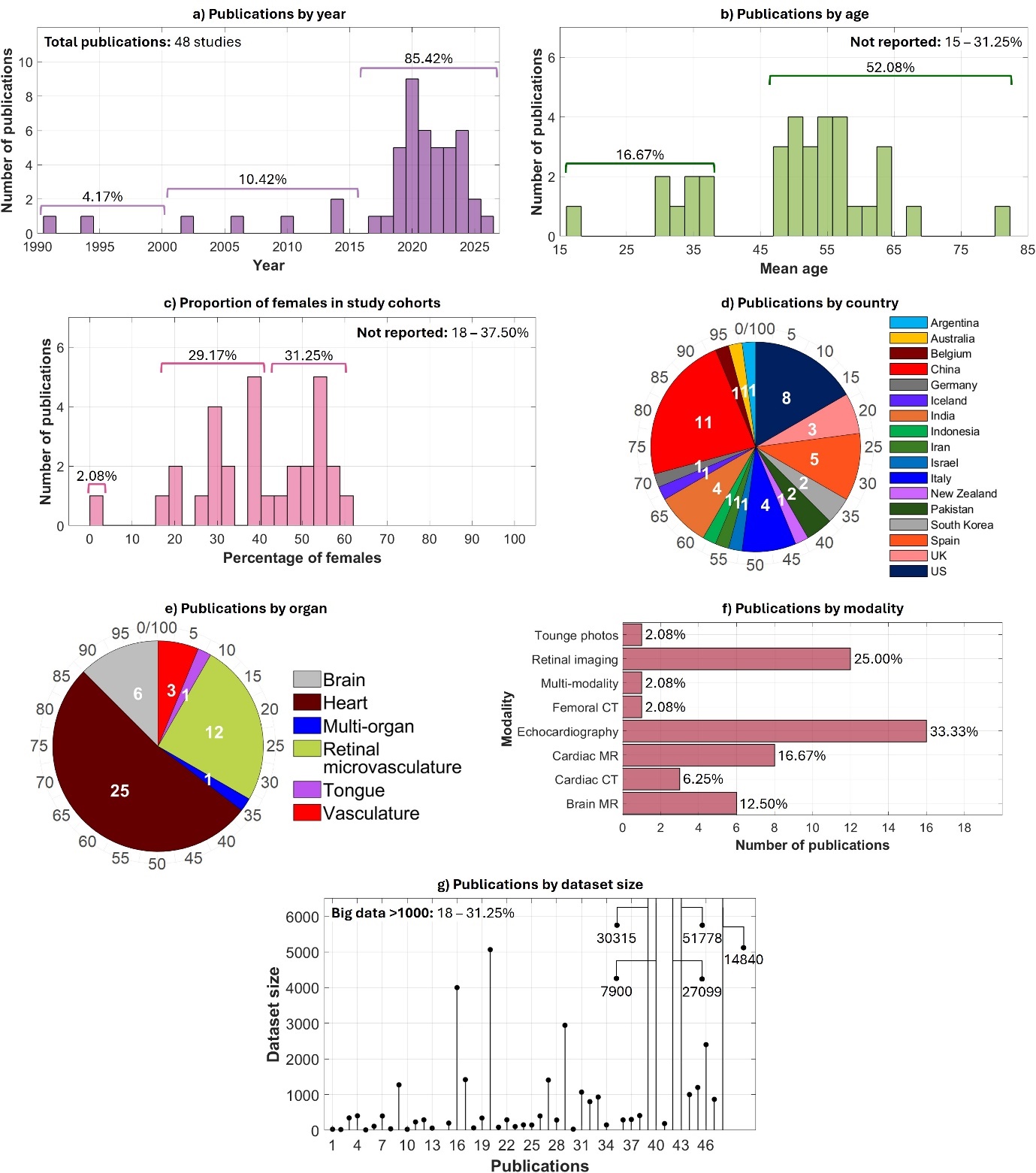
**Figure S1. Overall summary of the population included in the selected studies and type of used data.** A total of 48 studies were included in this systematic review. **a)** Publications by year revealed that the range of studies covered was between 1991 and 2026. **b)** Summary of publications by age was performed for studies with available data. **c)** Sex-based analysis was performed as well revealing few studies considering balanced number of females and males. **d)** 17 countries have led the research in these studies by correspondence/first authorship. Publications were further divided by: **e)** targeted organ for investigation and **f)** type of modality. **g)** Various datasets were used and varied between being small or big data. Big data is referred to as having more than 1,000 subjects or samples. CT: computed tomography; MR: magnetic resonance.


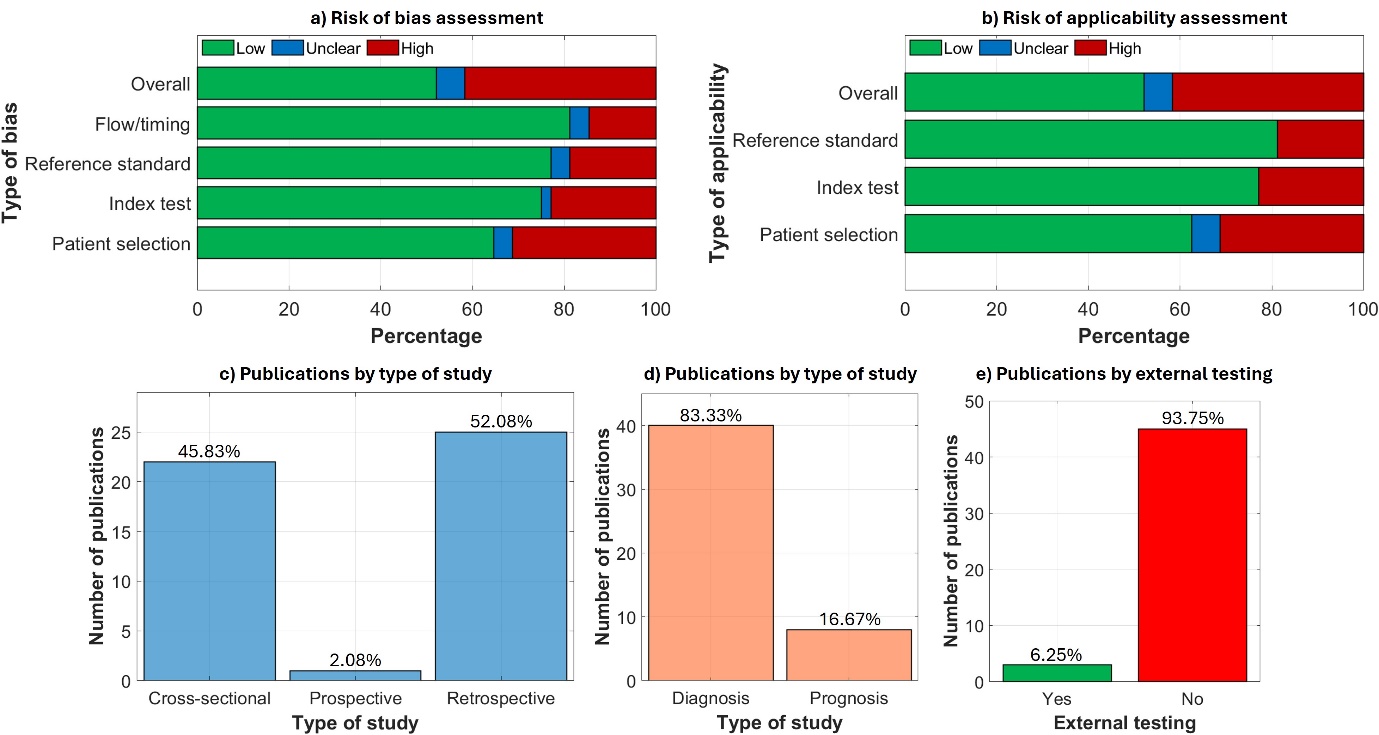
**Figure S2. Overall characterisation of included studies based on bias assessment and study type.** A total of 48 studies were included in this systematic review. Summary of studies was based on: The assessment of the risk of **a)** bias and **b)** applicability performed by taking into consideration flow/timing, reference standard, index test, and patient selection. **c)** Type of study design being cross-sectional, prospective, or retrospective, **d)** type of study clinically being diagnostic or prognostic, and **e)** whether an external validation was performed or not.


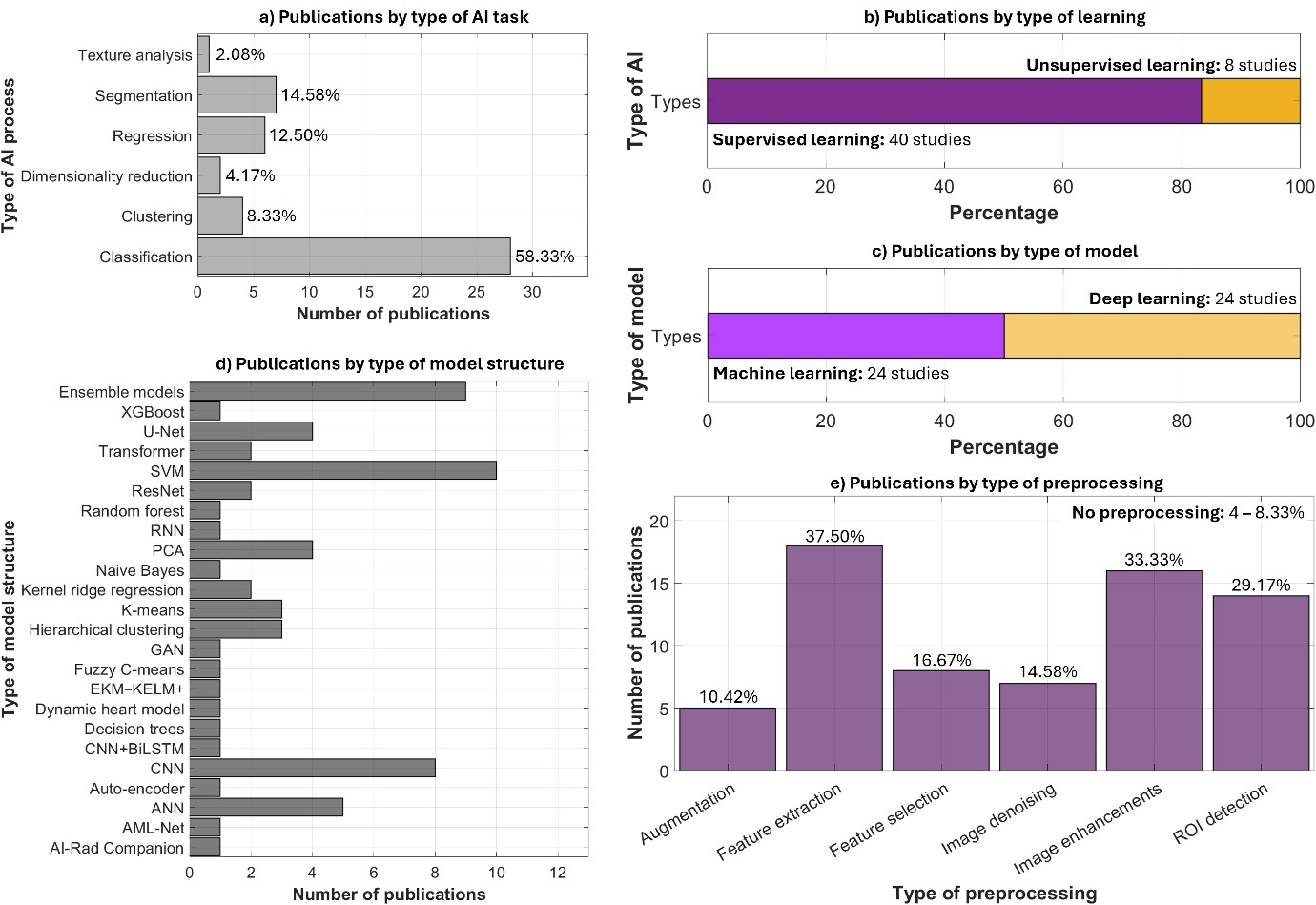
**Figure S3. Overall characterisation of included studies based on artificial intelligence approaches.** A total of 48 studies were included in this systematic review. Summary of studies was based on: **a)** Studies were divided based on the task of the AI algorithm, **b)** type of learning, and **c)** type of modelling. **d)** A thorough summary of the structure used when building the models is provided alongside **e)** the type of preprocessing procedures performed across studies.  Here, the number of studies may be repeated if more than two preprocessing steps were performed. XGBoost: eXtreme gradient boosting; SVM: support vector machine; ResNet: residual network; RNN: recurrent neural network; PCA: principal component analysis; GAN: generative adversarial network; EKM-KELM+: empirical kernel mapping-based kernel extreme learning machine plus; CNN-BiLSTIM: convolutional neural network Bi-directional long short-term memory; ANN: artificial neural network; ROI: region-of-interest.


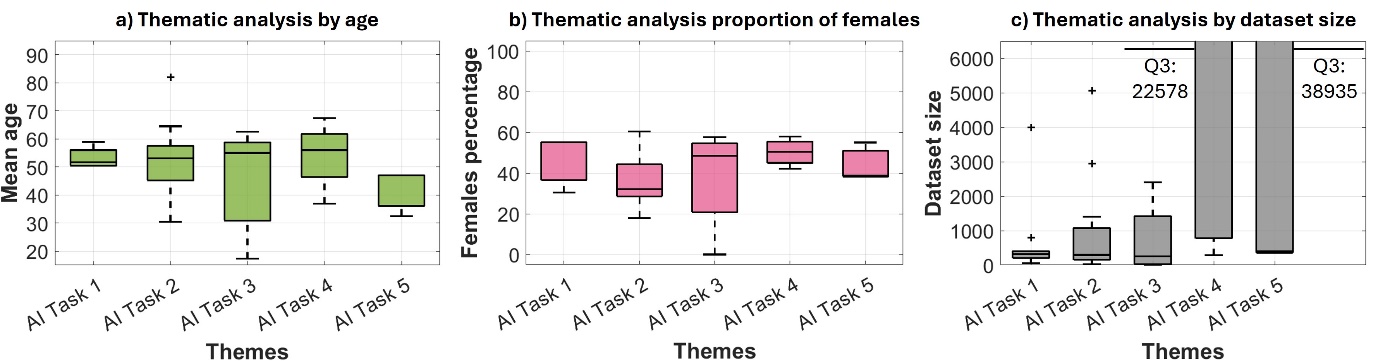
**Figure S4. Overall thematic analysis of AI Tasks.** A total of 48 studies were included in this systematic review. Studies were divided based on the five thematic AI tasks and analysed based on: **a)** mean age, **b)** proportion of females, and **c)** dataset size.
