# Supplementary material for: Applications of artificial intelligence and computational approaches to imaging for hypertension identification, phenotyping, and outcome prediction: a systematic review
Source: Eur Heart J Digit Health. 2026 Apr 20;7(4):ztag063. doi: 10.1093/ehjdh/ztag063 (PMC13175042; doi:10.1093/ehjdh/ztag063)
Supplement: ztag063_Supplementary_Data [file ztag063_supplementary_data.zip › Supplemental_tables_FINAL.docx]

**Table S1. Search strategies for Ovid/MEDLINE**

| No | Search queries for Ovid/MEDLINE, n = 164 [10/05/2023] |
| --- | --- |
| #1 | exp hypertension/ OR "high blood pressure".mp OR hypertensi*.tw,kf. OR ((high* OR rais* OR elevat* OR increas*) ADJ3 (systolic OR diastolic OR arterial OR pulse OR blood) ADJ3 pressure*).tw,kf. NOT pulmonary |
| #2 | exp "artificial intelligence"/ or (("artificial intelligence" or "machine learning" or "deep learning" or "supervised" or "unsupervised" or "reinforcement" or "neural network*" or "naive bayes" or "decision tree" or "random forest" or "support vector machine" or "k-nearest neighbour" or "linear discriminant analysis") adj3 (classifier or regression or classification or clustering)).tw,kf. |
| #3 | exp "computed tomography"/ or exp "magnetic resonance imaging"/ or exp ultrasonography/ or exp "pulse wave analysis"/ or ("CT imaging" or CT or X-ray or "magnetic resonance" or "MR imaging" or MRI or "cardiac MR*" or "cardiovascular MR*" or "diffusion tensor imaging" or "diffusion weight* imaging" or "diffusion imaging" or echocardiograph* or cardioechograph* or echocardiogram or doppler or "speckle tracking" or "pulse* wave velocity" or "cardiac structure*" or "myocardial structure*" or "cardiac function*" or "myocardial function*" or "intima media thickness" or "flow mediated dilatation" or microvascula* or microvessel* or macrovascula* or macrovessel*).tw,kf. or (ultraso* adj3 (brain or heart or cardi* or myocard* or ventric* or kidney* or nephr* or renal or vascul* or vessel*)).tw,kf. |
| #4 | #1 AND #2 AND #3 |

**Table S2. Search strategies for Ovid/EMBASE**

| No | Search queries for EMBASE, n = 392 [10/05/2023] |
| --- | --- |
| #1 | exp hypertension/ OR "high blood pressure".mp. OR hypertensi*.tw,kf. OR ((high* OR rais* OR elevat* OR increas* ) ADJ3 (systolic OR diastolic OR arterial OR pulse OR blood ) ADJ3 pressure* ).tw,kf. NOT pulmonary |
| #2 | exp "artificial intelligence"/ OR (("artificial intelligence" OR "machine learning" OR "deep learning" OR supervised OR unsupervised OR reinforcement OR "neural network*" OR "naive bayes" OR "decision tree" OR "random forest" OR "support vector machine" OR "k-nearest neighbour" OR "linear discriminant analysis" ) ADJ3 (classifier OR regression OR classification OR clustering )).tw,kf. |
| #3 | exp "computed tomography"/ OR exp "magnetic resonance imaging"/ OR exp ultrasonography/ OR exp "pulse wave analysis"/ OR ("CT imaging" OR CT OR X-ray OR "magnetic resonance" OR "MR imaging" OR MRI OR "cardiac MR*" OR "cardiovascular MR*" OR "diffusion tensor imaging" OR "diffusion weight* imaging" OR "diffusion imaging" OR echocardiograph* OR cardioechograph* OR echocardiogram OR doppler OR "speckle tracking" OR "pulse* wave velocity" OR "cardiac structure*" OR "myocardial structure*" OR "cardiac function*" OR "myocardial function*" OR "intima media thickness" OR "flow mediated dilatation" OR microvascula* OR microvessel* OR macrovascula* OR macrovessel* ).tw,kf. OR (ultraso* ADJ3 (brain OR heart OR cardi* OR myocard* OR ventric* OR kidney* OR nephr* OR renal OR vascul* OR vessel* )).tw,kf. |
| #4 | #1 AND #2 AND #3 |

**Table S3. Search strategies for Scopus**

| No | Search queries for Scopus, n = 46 [10/05/2023] |
| --- | --- |
| #1 | INDEXTERMS(hypertension) OR TITLE-ABS-KEY("high blood pressure") OR TITLE-ABS-KEY(hypertensi*) OR TITLE-ABS-KEY((high* OR rais* OR elevat* OR increas* ) W/3 (systolic OR diastolic OR arterial OR pulse OR blood ) W/3 pressure* ) NOT pulmonary |
| #2 | INDEXTERMS("artificial intelligence") OR TITLE-ABS-KEY(("artificial intelligence" OR "machine learning" OR "deep learning" OR supervised OR unsupervised OR reinforcement OR "neural network*" OR "naive bayes" OR "decision tree" OR "random forest" OR "support vector machine" OR "k-nearest neighbour" OR "linear discriminant analysis" ) W/3 (classifier OR regression OR classification OR clustering )) |
| #3 | INDEXTERMS("computed tomography") OR INDEXTERMS("magnetic resonance imaging") OR INDEXTERMS(ultrasonography) OR INDEXTERMS("pulse wave analysis") OR TITLE-ABS-KEY("CT imaging" OR CT OR X-ray OR "magnetic resonance" OR "MR imaging" OR MRI OR "cardiac MR*" OR "cardiovascular MR*" OR "diffusion tensor imaging" OR "diffusion weight* imaging" OR "diffusion imaging" OR echocardiograph* OR cardioechograph* OR echocardiogram OR doppler OR "speckle tracking" OR "pulse* wave velocity" OR "cardiac structure*" OR "myocardial structure*" OR "cardiac function*" OR "myocardial function*" OR "intima media thickness" OR "flow mediated dilatation" OR microvascula* OR microvessel* OR macrovascula* OR macrovessel* ) OR TITLE-ABS-KEY(ultraso* W/3 (brain OR heart OR cardi* OR myocard* OR ventric* OR kidney* OR nephr* OR renal OR vascul* OR vessel* )) |
| #4 | #1 AND #2 AND #3 |

**Table S4. Search strategies for PubMed**

| No | Search queries for PubMed, n = 392 [10/05/2023] |
| --- | --- |
| #1 | hypertension[Mesh] OR "high blood pressure"[tw] OR hypertensi*[tiab] OR ((high*[tiab] OR rais*[tiab] OR elevat*[tiab] OR increas*[tiab]) AND (systolic[tiab] OR diastolic[tiab] OR arterial[tiab] OR pulse[tiab] OR blood[tiab]) AND pressure*[tiab]) NOT pulmonary |
| #2 | "artificial intelligence"[Mesh] OR (("artificial intelligence"[tiab] OR "machine learning"[tiab] OR "deep learning"[tiab] OR supervised[tiab] OR unsupervised[tiab] OR reinforcement[tiab] OR "neural network*"[tiab] OR "naive bayes"[tiab] OR "decision tree"[tiab] OR "random forest"[tiab] OR "support vector machine"[tiab] OR "k-nearest neighbour"[tiab] OR "linear discriminant analysis"[tiab]) AND (classifier[tiab] OR regression[tiab] OR classification[tiab] OR clustering[tiab])) |
| #3 | "computed tomography"[Mesh] OR "magnetic resonance imaging"[Mesh] OR ultrasonography[Mesh] OR "pulse wave analysis"[Mesh] OR ("CT imaging"[tiab] OR CT[tiab] OR X-ray[tiab] OR "magnetic resonance"[tiab] OR "MR imaging"[tiab] OR MRI[tiab] OR "cardiac MR*"[tiab] OR "cardiovascular MR*"[tiab] OR "diffusion tensor imaging"[tiab] OR "diffusion weight* imaging"[tiab] OR "diffusion imaging"[tiab] OR echocardiograph*[tiab] OR cardioechograph*[tiab] OR echocardiogram[tiab] OR doppler[tiab] OR "speckle tracking"[tiab] OR "pulse* wave velocity"[tiab] OR "cardiac structure*"[tiab] OR "myocardial structure*"[tiab] OR "cardiac function*"[tiab] OR "myocardial function*"[tiab] OR "intima media thickness"[tiab] OR "flow mediated dilatation"[tiab] OR microvascula*[tiab] OR microvessel*[tiab] OR macrovascula*[tiab] OR macrovessel*[tiab]) OR (ultraso*[tiab] AND (brain[tiab] OR heart[tiab] OR cardi*[tiab] OR myocard*[tiab] OR ventric*[tiab] OR kidney*[tiab] OR nephr*[tiab] OR renal[tiab] OR vascul*[tiab] OR vessel*[tiab])) |
| #4 | #1 AND #2 AND #3 |

**Table S5. Search strategies for IEEE Xplore**

| No | Search queries for IEEE Xplore, n = 314 [10/05/2023] |
| --- | --- |
| #1 | (hypertension OR "high blood pressure" NOT pulmonary) |
| #2 | ("artificial intelligence" OR "machine learning" OR "deep learning" OR "supervised" OR "unsupervised" OR "reinforcement" OR "neural network" OR "naive bayes" OR "decision tree" OR "random forest" OR "support vector machine" OR "k-nearest neighbour" OR "linear discriminant analysis" OR classifier OR regression OR classification OR clustering) |
| #3 | ("medical imaging" OR imaging OR "computed tomography" OR "magnetic resonance imaging" OR ultrasonography OR "pulse wave analysis" OR "CT imaging" OR CT OR X-ray OR "magnetic resonance" OR "MR imaging" OR MRI OR "cardiac MR" OR "cardiovascular MR" OR "diffusion tensor imaging" OR "diffusion weighted imaging" OR "diffusion imaging" OR echocardiography OR cardioechography OR echocardiogram OR doppler OR "speckle tracking" OR "pulse wave velocity" OR "cardiac structure" OR "myocardial structure" OR "cardiac function" OR "myocardial function" OR "intima media thickness" OR "flow mediated dilatation" OR microvascular OR microvessel OR macrovascular OR macrovessels OR ultrasound) |
| #4 | #1 AND #2 AND #3 |

**Table S6. Search strategies for CINAHL**

| No | Search queries for CINAHL, n = 64 [10/05/2023] |
| --- | --- |
| #1 | (MH hypertension+) OR "high blood pressure" OR (TI hypertensi* OR AB hypertensi* OR SU hypertensi*) OR (((TI high* OR AB high* OR SU high*) OR (TI rais* OR AB rais* OR SU rais*) OR (TI elevat* OR AB elevat* OR SU elevat*) OR (TI increas* OR AB increas* OR SU increas*)) N3 ((TI systolic OR AB systolic OR SU systolic) OR (TI diastolic OR AB diastolic OR SU diastolic) OR (TI arterial OR AB arterial OR SU arterial) OR (TI pulse OR AB pulse OR SU pulse) OR (TI blood OR AB blood OR SU blood)) N3 (TI pressure* OR AB pressure* OR SU pressure*)) NOT pulmonary |
| #2 | (MH "artificial intelligence+") OR (((TI "artificial intelligence" OR AB "artificial intelligence" OR SU "artificial intelligence") OR (TI "machine learning" OR AB "machine learning" OR SU "machine learning") OR (TI "deep learning" OR AB "deep learning" OR SU "deep learning") OR (TI supervised OR AB supervised OR SU supervised) OR (TI unsupervised OR AB unsupervised OR SU unsupervised) OR (TI reinforcement OR AB reinforcement OR SU reinforcement) OR (TI "neural network*" OR AB "neural network*" OR SU "neural network*") OR (TI "naive bayes" OR AB "naive bayes" OR SU "naive bayes") OR (TI "decision tree" OR AB "decision tree" OR SU "decision tree") OR (TI "random forest" OR AB "random forest" OR SU "random forest") OR (TI "support vector machine" OR AB "support vector machine" OR SU "support vector machine") OR (TI "k-nearest neighbour" OR AB "k-nearest neighbour" OR SU "k-nearest neighbour") OR (TI "linear discriminant analysis" OR AB "linear discriminant analysis" OR SU "linear discriminant analysis")) N3 ((TI classifier OR AB classifier OR SU classifier) OR (TI regression OR AB regression OR SU regression) OR (TI classification OR AB classification OR SU classification) OR (TI clustering OR AB clustering OR SU clustering))) |
| #3 | (MH "computed tomography+") OR (MH "magnetic resonance imaging+") OR (MH ultrasonography+) OR (MH "pulse wave analysis+") OR ((TI "CT imaging" OR AB "CT imaging" OR SU "CT imaging") OR (TI CT OR AB CT OR SU CT) OR (TI X-ray OR AB X-ray OR SU X-ray) OR (TI "magnetic resonance" OR AB "magnetic resonance" OR SU "magnetic resonance") OR (TI "MR imaging" OR AB "MR imaging" OR SU "MR imaging") OR (TI MRI OR AB MRI OR SU MRI) OR (TI "cardiac MR*" OR AB "cardiac MR*" OR SU "cardiac MR*") OR (TI "cardiovascular MR*" OR AB "cardiovascular MR*" OR SU "cardiovascular MR*") OR (TI "diffusion tensor imaging" OR AB "diffusion tensor imaging" OR SU "diffusion tensor imaging") OR (TI "diffusion weight* imaging" OR AB "diffusion weight* imaging" OR SU "diffusion weight* imaging") OR (TI "diffusion imaging" OR AB "diffusion imaging" OR SU "diffusion imaging") OR (TI echocardiograph* OR AB echocardiograph* OR SU echocardiograph*) OR (TI cardioechograph* OR AB cardioechograph* OR SU cardioechograph*) OR (TI echocardiogram OR AB echocardiogram OR SU echocardiogram) OR (TI doppler OR AB doppler OR SU doppler) OR (TI "speckle tracking" OR AB "speckle tracking" OR SU "speckle tracking") OR (TI "pulse* wave velocity" OR AB "pulse* wave velocity" OR SU "pulse* wave velocity") OR (TI "cardiac structure*" OR AB "cardiac structure*" OR SU "cardiac structure*") OR (TI "myocardial structure*" OR AB "myocardial structure*" OR SU "myocardial structure*") OR (TI "cardiac function*" OR AB "cardiac function*" OR SU "cardiac function*") OR (TI "myocardial function*" OR AB "myocardial function*" OR SU "myocardial function*") OR (TI "intima media thickness" OR AB "intima media thickness" OR SU "intima media thickness") OR (TI "flow mediated dilatation" OR AB "flow mediated dilatation" OR SU "flow mediated dilatation") OR (TI microvascula* OR AB microvascula* OR SU microvascula*) OR (TI microvessel* OR AB microvessel* OR SU microvessel*) OR (TI macrovascula* OR AB macrovascula* OR SU macrovascula*) OR (TI macrovessel* OR AB macrovessel* OR SU macrovessel*)) OR ((TI ultraso* OR AB ultraso* OR SU ultraso*) N3 ((TI brain OR AB brain OR SU brain) OR (TI heart OR AB heart OR SU heart) OR (TI cardi* OR AB cardi* OR SU cardi*) OR (TI myocard* OR AB myocard* OR SU myocard*) OR (TI ventric* OR AB ventric* OR SU ventric*) OR (TI kidney* OR AB kidney* OR SU kidney*) OR (TI nephr* OR AB nephr* OR SU nephr*) OR (TI renal OR AB renal OR SU renal) OR (TI vascul* OR AB vascul* OR SU vascul*) OR (TI vessel* OR AB vessel* OR SU vessel*))) |
| #4 | #1 AND #2 AND #3 |

**Table S7. Search strategies for Web of Science**

| No | Search queries for Web of Science, n = 233 [10/05/2023] |
| --- | --- |
| #1 | ALL=hypertension OR ALL="high blood pressure" OR TS=hypertensi* OR TS=(((high* OR rais* OR elevat* OR increas*) NEAR/3 (systolic OR diastolic OR arterial OR pulse OR blood) NEAR/3 pressure*) NOT pulmonary) |
| #2 | ALL="artificial intelligence" OR TS=(("artificial intelligence" OR "machine learning" OR "deep learning" OR supervised OR unsupervised OR reinforcement OR "neural network*" OR "naive bayes" OR "decision tree" OR "random forest" OR "support vector machine" OR "k-nearest neighbour" OR "linear discriminant analysis" ) NEAR/3 (classifier OR regression OR classification OR clustering )) |
| #3 | ALL="computed tomography" OR ALL="magnetic resonance imaging" OR ALL=ultrasonography OR ALL="pulse wave analysis" OR TS=("CT imaging" OR CT OR X-ray OR "magnetic resonance" OR "MR imaging" OR MRI OR "cardiac MR*" OR "cardiovascular MR*" OR "diffusion tensor imaging" OR "diffusion weight* imaging" OR "diffusion imaging" OR echocardiograph* OR cardioechograph* OR echocardiogram OR doppler OR "speckle tracking" OR "pulse* wave velocity" OR "cardiac structure*" OR "myocardial structure*" OR "cardiac function*" OR "myocardial function*" OR "intima media thickness" OR "flow mediated dilatation" OR microvascula* OR microvessel* OR macrovascula* OR macrovessel* ) OR TS=(ultraso* NEAR/3 (brain OR heart OR cardi* OR myocard* OR ventric* OR kidney* OR nephr* OR renal OR vascul* OR vessel* )) |
| #4 | #1 AND #2 AND #3 |

**Table S8. Risk of bias and applicability assessment for studies included in the review.**

| **Study** | **Risk of bias (ROB)** | | | | **Applicability** | | | **Overall** | |
| --- | --- | --- | --- | --- | --- | --- | --- | --- | --- |
|  | Patient selection | Index test | Reference standard | Flow and Timing | Patient selection | Index test | Reference standard | ROB | Applicability |
| **AI Task 1**  **Identifying hypertension or its stages** | | | | | | | | | |
| Morales et al.^1^ | • | • | • | • | • | • | • | • | • |
| Lo et al.^2^ | • | • | • | • | • | • | • | • | • |
| Yu et al.^3^ | • | • | • | • | • | • | • | • | • |
| Kandil et al.^4^ | • | • | • | • | • | • | • | • | • |
| Kandil et al.^5^ | • | • | • | • | • | • | • | • | • |
| Kandil et al.^6^ | • | • | • | • | • | • | • | • | • |
| Peng et al.^7^ | • | • | • | • | • | • | • | • | • |
| Cetin et al.^8^ | • | • | • | • | • | • | • | • | • |
| Wang et al.^9^ | • | • | • | • | • | • | • | • | • |
| Karki et al.^10^ | • | • | • | • | • | • | • | • | • |
| **AI Task 2**  **Differentiating hypertension from other diseases** | | | | | | | | | |
| Rose et al.^11^ | • | • | • | • | • | • | • | • | • |
| Koduri et al.^12^ | • | • | • | • | • | • | • | • | • |
| Azhari *et al.*^13^ | • | • | • | • | • | • | • | • | • |
| Neisius et al.^14^ | • | • | • | • | • | • | • | • | • |
| Cetin et al.^15^ | • | • | • | • | • | • | • | • | • |
| Vidal-Sospedra et al.^16^ | • | • | • | • | • | • | • | • | • |
| Yu et al.^17^ | • | • | • | • | • | • | • | • | • |
| Sabovčik et al.^18^ | • | • | • | • | • | • | • | • | • |
| Shi et al.^19^ | • | • | • | • | • | • | • | • | • |
| Forghani et al.^20^ | • | • | • | • | • | • | • | • | • |
| Barbieri et al.^21^ | • | • | • | • | • | • | • | • | • |
| Hwang et al.^22^ | • | • | • | • | • | • | • | • | • |
| Xu et al.^23^ | • | • | • | • | • | • | • | • | • |
| Zhang et al.^24^ | • | • | • | • | • | • | • | • | • |
| Diao et al.^25^ | • | • | • | • | • | • | • | • | • |
| Wang et al.^26^ | • | • | • | • | • | • | • | • | • |
| Wang et al.^27^ | • | • | • | • | • | • | • | • | • |
| Moon et al.^28^ | • | • | • | • | • | • | • | • | • |
| Recenti et al.^29^ | • | • | • | • | • | • | • | • | • |
| **AI Task 3**  **Characterising target end-organ changes** | | | | | | | | | |
| Kaupp et al.^30^ | • | • | • | • | • | • | • | • | • |
| Khitran et al.^31^ | • | • | • | • | • | • | • | • | • |
| Ahmad et al.^32^ | • | • | • | • | • | • | • | • | • |
| Kiruthika et al.^33^ | • | • | • | • | • | • | • | • | • |
| Dai et al.^34^ | • | • | • | • | • | • | • | • | • |
| Bhimavarapu et al.^35^ | • | • | • | • | • | • | • | • | • |
| Triwijoyo et al.^36^ | • | • | • | • | • | • | • | • | • |
| Raghavendra et al.^37^ | • | • | • | • | • | • | • | • | • |
| Alsharqi et al.^38^ | • | • | • | • | • | • | • | • | • |
| Pessana et al.^39^ | • | • | • | • | • | • | • | • | • |
| Alkhodari et al.^40^ | • | • | • | • | • | • | • | • | • |
| **AI Task 4**  **Identifying phenotypes and hypertension subgroups** | | | | | | | | | |
| Yang et al.^41^ | • | • | • | • | • | • | • | • | • |
| Katz et al.^42^ | • | • | • | • | • | • | • | • | • |
| Loncaric et al.^43^ | • | • | • | • | • | • | • | • | • |
| Rauseo et al.^44^ | • | • | • | • | • | • | • | • | • |
| **AI Task 5**  **Defining risk of future hypertension-related events** | | | | | | | | | |
| Squirrell et al.^45^ | • | • | • | • | • | • | • | • | • |
| Giovanna et al.^46^ | • | • | • | • | • | • | • | • | • |
| Viazzi et al.^47^ | • | • | • | • | • | • | • | • | • |
| **Other additional**  **applications** | | | | | | | | | |
| Jana et al.^48^ | • | • | • | • | • | • | • | • | • |

A total of 48 studies were included in this systematic review. Red dots indicate high risk (label: -1). Green dots indicate low risk (label: 1). Grey dots indicate unclear risk (label: 0).

**Table S9. Summary of studies based on organ details in hypertension observed by imaging and AI.**

| Study | Year | Organ | Type of modality | Type of hypertension | Findings |
| --- | --- | --- | --- | --- | --- |
| AI Task 1  Identifying hypertension or its stages | | | | | |
| Morales et al.^1^ | 2014 | Retinal microvasculature | Retinal imaging | Hypertensive retinopathy | - Segmentation algorithm was able of segmenting vessels which increased accuracy by 20% compared with visual inspection |
| Lo et al.^2^ | 2021 |  | Retinal imaging | Hypertensive retinopathy | - Fundus images during hypertension had brighter vessels with less similarity to anchor images |
| Yu et al.^3^ | 2019 | Brain | Brain MR | Arterial hypertension | - More reduction of brain tissue due to hypertension  - Thickness volume reductions were found in frontal, occipital, limbic, parietal, and temporal lobes of hypertensive patients  - In insula and sub-cortical grey nuclei, CSF volume increased with BP |
| Kandil et al.^4^ | 2019 |  | Brain MR | Arterial hypertension | - Brain vascular diameter and tortuosity distributions were correlated with the increase in blood pressure |
| Kandil et al.^5^ | 2020 |  | Brain MR | Arterial hypertension | - The high performance was linked with alterations that affect cerebrovascular structures |
| Kandil et al.^6^ | 2020 |  | Brain MR | Arterial hypertension | - Systolic blood pressure was more predictive than diastolic blood pressure relative to brain vasculature alterations |
| Peng et al.^7^ | 2020 |  | Brain MR | Arterial hypertension | - Grade 2 hypertension showed structural changes in frontal and temporal lobes  - Grade 3 hypertension showed changes in frontal, parietal, and temporal lobes  - Grade 4 hypertension showed changes in frontal, temporal, parietal, and subcortical lobes |
| Cetin et al.^8^ | 2019 | Heart | Cardiac MR | Arterial hypertension | - Intensity and texture radiomics were impactful more than geometry and size of myocardium to identify cardiac alterations in hypertension  - Large area emphasis, short run emphasis, and grey level non-uniformity were important radiomics values |
| Wang et al.^9^ | 2022 | Heart | Cardiac CT | Masked hypertension | - Aortic diameter at the position of mid descending was important  - Aorta measured by AI was a risk factor for adverse BP status |
| Karki et al.^10^ | 2020 | Tongue | Tongue images | Arterial hypertension | - Tongue was useful in classifying hypertension cases with liver abnormalities |
| AI Task 2  Differentiating hypertension from other diseases | | | | | |
| Rose et al.^11^ | 2023 | Retinal microvasculature | Retinal imaging | Hypertensive retinopathy | - The predicted HT cases had circular white spots on the retina indicating abnormality  - Vessels were brighter in colour and larger in diameter |
| Koduri et al.^12^ | 2024 |  | Retinal imaging | Hypertensive retinopathy | - HR cases had narrower arterioles compared to diabetic ones |
| Azhari *et al.*^13^ | 1991 | Heart | Cardiac CT | Arterial hypertension | - Healthy LV was less dilated/spherical compared with diseased hearts  - Smaller heart geometrical regularity index in diseased hearts  - Overall structural abnormalities of heart chamber in diseased hearts |
| Neisius et al.^14^ | 2019 |  | Cardiac MR | Arterial hypertension | - Global myocardial native T1 values were significantly lower compared with HCM  - Texture features of LBP-28 and RLN-135 played an important role in discriminating between HCM and hypertension |
| Cetin et al.^15^ | 2020 |  | Cardiac MR | Arterial hypertension | - Surface area to volume ratio was smaller in hypertension reflecting concentric LV hypertrophy  - Spherical disproportion of the myocardium at end-diastole was lower with high cholesterol |
| Vidal-Sospedra et al.^16^ | 2020 |  | Cardiac MR | Arterial hypertension | - Hypertensive cardiomyopathy was correlated with more clutter in image and heterogeneity in pixels |
| Yu et al.^17^ | 2020 |  | Echocardiography | Arterial hypertension | - Texture features of EtBrt, Std, and CoV had high diagnostic power and reproducibility  - The myocardium appeared heterogenous with histological alterations  - LV remodelling was associated with different levels of myocardial hyperplasia, myocardial disarray, and interstitial fibrosis |
| Sabovčik et al.^18^ | 2021 |  | Echocardiography | Arterial hypertension | - BMI and BP features were associate with LV structural and Doppler diastolic indexes |
| Shi et al.^19^ | 2021 |  | Cardiac MR | Arterial hypertension | - Radial, circumferential, and longitudinal strain parameters identified hypertension  - Only longitudinal strain parameters were significantly different between HCM and HHD |
| Forghani et al.^20^ | 2021 |  | Echocardiography | Arterial hypertension | The combination of echocardiography and ECG information leads to performance improvements |
| Barbieri et al.^21^ | 2022 |  | Echocardiography | Arterial hypertension | - DHM-based LA/LV showed high odds ratio with CVDs (>3)  - LAVi min, LAEF, LVEF, and LVGFI were highly associated with HT |
| Hwang et al.^22^ | 2022 |  | Echocardiography | Arterial hypertension | - LVWT was >12 mm for both HHD and ALCA, and the mean LVWT did not differ between the two groups  - Important regions for the predictions of HT: ascending aorta on PLAX views, RV insertion site on PSAX views, and RV apex and LV inferior/inferolateral wall on apical views |
| Xu et al.^23^ | 2022 |  | Echocardiography | Arterial hypertension | - Time/frequency domain LV contractility features were important for differentiating HT and UCM  - No decline in LV diastolic function in HT  - IVS thickness was lower in HT and UCM compared to HCM |
| Zhang et al.^24^ | 2023 |  | Echocardiography | Arterial hypertension | - HT cases had the lowest IVS, LVPW, and LVMI  - First-order quartile deviation, total energy, and three original grey level matrix features were important to identify hypertension |
| Diao et al.^25^ | 2023 |  | Cardiac MR | Arterial hypertension | - LV myocardium thickness was the least in HT  - The morphology/motion features of the LV myocardium during a cardiac cycle had the highest accuracy |
| Wang et al.^26^ | 2024 |  | Cardiac MR | Arterial hypertension | - DL was superior to T1 features in identifying HT  - DL was comparable to radiomics in performance  - DL based on T1 mapping was the optimal selection |
| Wang et al.^27^ | 2024 |  | Cardiac MR | Arterial hypertension | - DL identified symmetric left ventricular hypertrophy as important for HT prediction  - LV dysfunctions were highly associated with HT  - 4-channel cine and SAX LGE were highly important |
| Moon et al.^28^ | 2025 |  | Echocardiography | Arterial hypertension | - Texture and myocardial thickness were influential in differentiating HHD  - harmonization-driven textures identified HCM |
| Recenti et al.^29^ | 2021 | Vasculature | Femoral CT | Arterial hypertension | - High importance of connective tissue parameters |
| AI Task 3  Characterising target end-organ changes | | | | | |
| Kaupp et al.^30^ | 1994 | Retinal microvasculature | Retinal imaging | Hypertensive retinopathy | - The tortuosity of retinal arteries in hypertension was 25% higher than normotensives  - Arteries diameter was significantly decreased |
| Khitran et al.^31^ | 2014 |  | Retinal imaging | Hypertensive retinopathy | - Retinal arteriovenous ratio was below 0.5 in hypertensives |
| Ahmad et al.^32^ | 2018 |  | Retinal imaging | Hypertensive retinopathy | - Arteries and veins were highly distinguishable in hypertensive retinopathy cases |
| Kiruthika et al.^33^ | 2019 |  | Retinal imaging | Hypertensive retinopathy | - Arteries and veins were highly distinguishable due to hypertension |
| Dai et al.^34^ | 2020 |  | Retinal imaging | Hypertensive retinopathy | - Areas around arterial/venous bifurcations helped in the prediction of hypertension  - Retinal arteriolar branching asymmetric ratios were correlated with mean arterial blood pressure  - Arteriolar/venular branching angle and venular branching asymmetry ratio were not related to blood pressure |
| Bhimavarapu et al.^35^ | 2024 |  | Retinal imaging | Hypertensive retinopathy | - Strong performance in images with thin blood vessels and arteries and those closest to optic nerve |
| Triwijoyo et al.^36^ | 2025 |  | Retinal imaging | Hypertensive retinopathy | - Retina texture, blood vessels, hard exudates, bleeding, and cotton wool spots were important |
| Raghavendra et al.^37^ | 2022 | Heart | Echocardiography | Arterial hypertension | - The SHELet and CWT transform features were important geometrical properties such as orientation, curvature of discontinuity, shape, edging, and contouring |
| Alsharqi et al.^38^ | 2023 |  | Echocardiography | Arterial hypertension | - Left atrial reservoir and conduit function drop with risk score  - Booster pump function increases initially and then reduces with severity  - LV volume was less as the disease advances with higher assigned scores  - Fully complaint aerobic exercise reduced the AI-based cardiac remodelling score |
| Pessana et al.^39^ | 2010 | Vasculature | Carotid ultrasound | Arterial hypertension | - Elasticity, viscosity, and inertial indexes were estimated similarly to ground-truth method |
| Alkhodari et al.^40^ | 2023 | Multi-organ | Cardiac MR, brain MR, and carotid ultrasound | Arterial hypertension | - Risk score was correlated with higher age, BMI, WMH, and IMT  - A non-linear relation was observed in  cholesterol level and LVS  - Gray matter volume and body impedance had negative linear correlation with risk scores |
| AI Task 4  Identifying phenotypes and hypertension subgroups | | | | | |
| Yang et al.^41^ | 2024 | Brain | Brain MR | Arterial hypertension | - Five HT subtypes were identified with distinct imaging patterns  - WMH was higher at two groups of advanced state with higher rates of diabetes  - No differences were observed in hyperlipidemia |
| Katz et al.^42^ | 2017 | Heart | Echocardiography | Arterial hypertension | - Two phenogroups were identified that varied significantly in function, structure, and mechanics  - A group had worse outcomes and abnormal LV mass, LVED, LVS, and LVWT  - This group had decreased absolute longitudinal strain |
| Loncaric et al.^43^ | 2021 |  | Echocardiography | Arterial hypertension | - Four major phenotypical groups were identified  - They were significantly different in fused E and A wave, septal/lateral mitral annular e’, mitral average, and LV function |
| Rauseo et al.^44^ | 2026 |  | Cardiac MR | Arterial hypertension | - Three clusters were identified related to lowest cardiac remodelling (best outcomes), metabolic syndrome, and highest atherosclerotic and cardiac damage |
| AI Task 5  Defining risk of future hypertension-related events | | | | | |
| Squirrell et al.^45^ | 2024 | Retinal microvasculature | Retinal imaging | Hypertensive retinopathy | - Narrower retinal arterioles and wider venules confer long-term risk of mortality due to ASCVD |
| Giovanna et al.^46^ | 2002 | Heart | Echocardiography | Arterial hypertension | - Target organ damage influences the classification of overall cardiovascular risk  - Organ damage is a multifactorial process caused by blood pressure, genetics, and hormonal/metabolic abnormalities  - Microalbuminuria was correlated with LVMI, LVH, and thicker IMT |
| Viazzi et al.^47^ | 2006 |  | Echocardiography | Arterial hypertension | - Higher estimated risk was linked with a reduction in creatinine clearance and eGFR  - Organ damage was associated with high estimate risk and reduced eGFR |
| Other additional  applications | | | | | |
| Jana et al.^48^ | 2020 | Vasculature | Doppler ultrasound | Arterial hypertension | - Brachial artery was most important in providing accurate cuff-less estimates for blood pressure |

A total of 48 studies were included in this systematic review. SBP: systolic blood pressure; DBP: diastolic blood pressure; BMI: body mass index; HR: hypertensive retinopathy; MR: magnetic resonance; CT: computed tomography; HT: hypertension; LA: left atrium; RV: right ventricle; LV: left ventricle; HCM: hypertrophic cardiomyopathy; HIP: hypertensive cardiomyopathy; AM: amyloidosis; UCM: uremic cardiomyopathy; HHD: hypertensive heart disease; LVH: left ventricular hypertrophy; LVED: left ventricular end-diastolic; LVS: left ventricular stroke; LVDD: left ventricular diastolic dysfunction; LGE: late gadolinium enhancement; ALCA: anomalous left coronary artery; LAVi: left atrial volume index; LAEF: left atrial ejection fraction; LVEF: left ventricular ejection fraction; LVGFI: left ventricular global function index; CWT: continuous wavelet transform; SHELet: Shearlet transform; WMA: wall motion abnormality; LVWT: left ventricular wall thickness; LVMI: left ventricular mass index; ESRD: end-stage renal disease; eGFR: estimated glomerular filtration rate; A/V ratio: arteriovenous ratio; HICH: Hypertensive intracerebral haemorrhage; PLAX: parasternal long axis view; PSAX: parasternal short axis view; CSF: cerebrospinal fluid; ASCVD : atherosclerotic cardiovascular disease; IMT: intima–media thickness; WMH: white matter hyperintensities; IVS: interventricular Septum; LVPW: left ventricular posterior wall; DHM: deformable heart model.

**Table S10. Summary of studies based on AI models and performance in identifying hypertension.**

| Study | Year | Type of learning | Modelling task | Type of supervision | Model information | Input data | Performance | Limitations |
| --- | --- | --- | --- | --- | --- | --- | --- | --- |
| AI Task 1  Identifying hypertension or its stages | | | | | | | | |
| Morales et al.^1^ | 2014 | Machine learning | Dimensionality  reduction and segmentation | Unsupervised | PCA, K-means | - Retinal images | - Accuracy: 94.17%  - TPF: 0.66  - FPF: 0.02 | - Not applicable when bifurcation angles are less than 5 per image |
| Cetin et al.^8^ | 2019 |  | Classification | Supervised | SVM | - Heart shape, size, intensity, and textural | - AUC: 0.76 | - No clinical interpretation of the results  - No considerations for other risk factors |
| Peng et al.^7^ | 2020 |  | Classification | Supervised | Kernel ridge regression | - Brain morphological features | - Accuracy: 95.6%  - Sensitivity: 96.8%  - Specificity: 94.5% | - The performance is based on the selected of HT grades  - Algorithm was not tested under severe conditions |
| Yu et al.^3^ | 2019 | Deep learning | Classification | Supervised | EKM–KELM+ | - Brain structural features  - Brain network features | - Accuracy: 95.2%  - Sensitivity: 97.1%  - Specificity: 93.1% | - Small sample size  - No selection of age groups  - Models were not interpreted properly |
| Kandil et al.^4^ | 2019 |  | Segmentation and classification | Supervised | CNN, SVM | - Raw brain images  - Brain vascular features | - DSC: 84.4%  - Accuracy: 90%  - AUC: 0.89 | - Small sample size  - No severe conditions |
| Kandil et al.^5^ | 2020 |  | Segmentation and classification | Supervised | CNN | - Raw brain images | - DSC: 84.3%  - Accuracy: 90.9%  - AUC: 0.91 | - No clinical validation  - No clinical interpretation of model decisions |
| Kandil et al.^6^ | 2020 |  | Segmentation and classification | Supervised | CNN | - Raw brain images | - Accuracy: 89.3% | - No clinical validation  - No clinical interpretation of model decisions |
| Karki et al.^10^ | 2020 |  | Classification | Supervised | CNN | - Raw tongue images | - Accuracy: 94.3% | - No interpretation of model  - Tongue could be a weak organ to reflect hypertension |
| Lo et al.^2^ | 2021 |  | Classification | Supervised | AML-Net | - Raw retinal images | - Accuracy: 93.8% | - Similarity does not always imply correct classification  - Network decisions were not interpreted  - No extensive clinical validation |
| Wang et al.^9^ | 2022 |  | Segmentation | Supervised | AI-Rad companion, auto-encoders | - Raw CT aorta images | - AUC: 0.78 | - Patients were not screened through home BP monitoring  - The algorithm failed to detect landmark due to defects in CT images |
| AI Task 2  Differentiating hypertension from other diseases | | | | | | | | |
| Azhari *et al.*^13^ | 1991 | Machine learning | Clustering | Unsupervised | Fuzzy C-means | - LV geometry features | - Accuracy: 85% - Four optimal clusters  (matched with clinical terms) | - Small sample size  - Simple clustering algorithm |
| Neisius et al.^14^ | 2019 |  | Classification | Supervised | SVM | - Texture features from native T1 maps | - Accuracy: 86.2%  - C-statistic: 0.820  - AUC: 0.82 | - Hypertension was defined by LVWT not hypertrophy  - No 3-dimensional spatial orientation was considered |
| Vidal-Sospedra et al.^16^ | 2020 |  | Classification | Supervised | SVM | - LV intensity and geometry features | - AUC: 0.91  - Sensitivity: 92.3%  - Specificity: 72.7% | - Small sample size  - No details on the clinical relation between prediction and features  - Dependent on LV only |
| Yu et al.^17^ | 2020 |  | Classification | Supervised | SVM | - TTE myocardial texture features | - Accuracy: 68%  - AUC: 0.7  - Sensitivity: 59%  - Specificity: 78% | - Histological and genetic evidence was lacking for diagnosis  - HCM cases had an LV wall over 15 mm thick |
| Cetin et al.^15^ | 2020 |  | Classification | Supervised | Ensemble learning | - Heart shape, size, intensity, and textural | - Accuracy: 68.2%  - AUC: 0.72 | - Validation was not enough  - No validation on the reproducibility of radiomics |
| Sabovčik et al.^18^ | 2021 |  | Classification | Supervised | Ensemble learning | - Clinical data, biochemical measurements, and ECG | - AUC: 0.88  - Sensitivity: 85.7%  - Specificity: 74.8% | - Small sample size  - Echocardiography was prone to errors  - Echocardiography was used only as a ground truth |
| Shi et al.^19^ | 2021 |  | Classification | Unsupervised | Random forest | - T1 maps and extracellular volume texture features | - AUC: 0.89 | - Small sample size  - Feature were not average across different directions  - No combination of multi-feature parametric map |
| Recenti et al.^29^ | 2021 |  | Classification | Supervised | Decision trees | - CT HU NTRA features | - Accuracy: 90.2%  - AUC: 0.98 | - No comparison with conventional methods  - No considerations for demographics of patients |
| Forghani et al.^20^ | 2021 |  | Classification | Supervised | SVM | - Echocardiography strain, strain rate, and ECG | Precision: 97.62%,  Sensitivity: 93.33%  F1 = 95.43% | - Small sample size  - Manual selection for initial kernels for speckle tracking of the apex |
| Zhang et al.^24^ | 2023 |  | Classification | Supervised | Ensemble learning | - Myocardial texture features  (B-mode ultrasound) | - Accuracy: 87.0 %  - AUC: 0.92  - Sensitivity: 81.0%  - Specificity: 91.0% | - Blood and urine tested were not performed  - Echo quality was affected by many factors  - Selectivity bias due to less LVH in patients |
| Rose et al.^11^ | 2023 |  | Classification | Supervised | SVM | - Retinal vessel color, shape, texture, size, and ridges | - Accuracy: 97.5%  - AUC: 0.75  - Sensitivity: 96.0% | - Small sample size  - ROC curve was not ideal  - Segmentation was based on intensity only not shape |
| Moon et al.^28^ | 2025 |  | Classification | Supervised | XGBoost | Conventional and harmonization-driven myocardial textures, and myocardial  geographic features | Sensitivity = 75.0%  F1 = 82.0% | - Focus on Korean ethnicity  - LVH was not comprehensive excluding other conditions (athlete heart) |
| Hwang et al.^22^ | 2022 | Deep learning | Classification | Supervised | CNN-LSTM | - Raw echocardiograms | - Accuracy: 92.3%  - AUC: 0.96 | - No considerations for LVH etiologies  - Excluded patients with severe cardiac/liver diseases  -Excluded images with overt echo abnormalities |
| Xu et al.^23^ | 2022 |  | Segmentation and classification | Supervised | U-Net | - Raw echocardiograms  - Echo intensity statistics and time/frequency features | - DSC: 0.84  - Accuracy: 84.3%  - AUC: 0.84  - Sensitivity: 77%  - Specificity: 99.8% | - Automatic segmentation model was subjective and operator-dependent  - Only investigated three primary causes of LVH  - Only focusing on the interventricular septum segmentation |
| Barbieri et al.^21^ | 2022 |  | Texture analysis | Supervised | Dynamic heart model | - LV function/structure features | NA | - Lack of history of factors affecting cardiac remodelling  - No considerations for LV/LA global longitudinal strain  - 2DE quantitative morphometric evaluation was not performed |
| Diao et al.^25^ | 2023 |  | Segmentation | Supervised | U-Net, RNN | - Raw MRI images  - Segmented myocardium regions | - Accuracy: 77.4%  - AUC: 0.98 | - Need more consideration for other LVH etiologies  - No usage of common clinical variables, *i.e.*, T1 |
| Wang et al.^26^ | 2024 |  | Classification | Supervised | ResNet | - Raw MRI images  - Segmented myocardium regions | - Accuracy: 73.7%  - AUC: 0.83  - Sensitivity: 76.9%  - Specificity: 66.7% | - Single center dataset  - Inclusion of only 2D diastolic phase  - Less interpretability of the model |
| Wang et al.^27^ | 2024 |  | Classification | Supervised | Swin transformer | - Raw MRI images | - Accuracy: 93.20%  - F1-score: 0.833  - AUC: 0.972 | - No details on the use of T1 or T2  - Complex or rare phenocopies were hardly distinguished |
| Koduri et al.^12^ | 2024 |  | Classification | Supervised | Dual vision transformer | - Raw retinal images | - Accuracy: 97.98%  - Sensitivity: 98%  - Precision: 98%  - F1-score: 97.9% | - Black-box predictions with no interpretation  - Small dataset with lack of additional testing |
| AI Task 3  Characterising target end-organ changes | | | | | | | | |
| Khitran et al.^31^ | 2014 | Machine learning | Classification | Supervised | Naïve-bayes, SVM | - Vessel intensity features | - Accuracy: 98%  - Sensitivity: 97%  - Arteriovenous ratio: 0.1-0.5 | - Performance is dependent on vessel segmentation  - Algorithm was not tested under severe conditions |
| Ahmad et al.^32^ | 2018 |  | Classification | Supervised | Ensemble learning | - Segmented retinal vasculature features | - Accuracy: 89.4%  - AUC: 0.94 | - Arteriovenous ratio was not reported  - Algorithm was not tested under severe conditions |
| Raghavendra et al.^37^ | 2022 |  | Classification | Supervised | Ensemble learning | - Echocardiogram features using different transforms | - Accuracy: 99.1 %  - Sensitivity: 100%  - Specificity: 100% | - No clinical validation  - Performance was not interpreted properly |
| Alsharqi et al.^38^ | 2023 |  | Regression | Unsupervised | Contrastive PCA | - Echocardiogram extracted features of the LV  (Doppler velocities and speckle tracking features) | - AUC: 0.98 | - Model had more subjects with low scores  - Single center datasets  - Some variables are not routinely acquired clinically |
| Alkhodari et al.^40^ | 2023 |  | Regression | Unsupervised | Contrastive PCA | - Features extracted from multiple modalities | - Accuracy: 78.6 %  - AUC: 0.87  - Sensitivity: 77.0%  - Specificity: 78.0% | - No further analysis of adverse outcomes  - Less interpretability of the model |
| Kaupp et al.^30^ | 1994 | Deep learning | Classification | Supervised | ANN | - Segmented vessels image | NA | - No performance was reported  - Small sample size  - No clinical validity of the proposed approach |
| Pessana et al.^39^ | 2010 |  | Regression | Supervised | ANN | - Carotid artery images | - Correlation: 0.96  - No significant difference in arterial diameter and mechanic indexes between | - Invariance in the estimated biomechanical parameters  - No changes to the posterior assessment of vascular walls |
| Kiruthika et al.^33^ | 2019 |  | Classification | Supervised | CNN, ANN | - Vessel textural, intensity, statistical, and width features | - Accuracy: 92.6%  - Sensitivity: 93.8% | - Prediction of condition is dependent on segmentation performance  - No severe conditions |
| Dai et al.^34^ | 2020 |  | Segmentation and classification | Supervised | U-Net, CNN | - Raw retinal images | - Accuracy: 60.9%  - AUC: 0.65 | - No clinical validation  - Prediction of condition is dependent on segmentation performance |
| Bhimavarapu et al.^35^ | 2024 |  | Classification | Supervised | CNN | - Raw retinal images | - Accuracy: 99.37%  - Sensitivity: 98.75%  - Specificity: 99.56% | - No validation on clinical dataset  - Small dataset |
| Triwijoyo et al.^36^ | 2025 |  | Classification | Supervised | CNN | - Raw retinal images | - Accuracy: 81.82%  - Sensitivity: 90%  - Specificity: 90% | - Manual labelling of HR stages based on AVR  - Individual had diabetic retinopathy besides HR |
| AI Task 4  Identifying phenotypes and hypertension subgroups | | | | | | | | |
| Katz et al.^42^ | 2017 | Machine learning | Clustering | Unsupervised | Hierarchical clustering | - Cardiac structure/function  - Demographic characteristics  - Serum/urine laboratory | - Bayesian criterion: -135000  - Two optimal clusters | - Lack of validation  - Limited variable set |
| Loncaric et al.^43^ | 2021 | Machine learning | Dimensionality  reduction | Unsupervised | Kernel ridge regression, K-means, Hierarchical clustering | - Full cardiac cycle deformation and velocity curves  - Aortic and mitral blood pool pulsed-wave Doppler  - Mitral annular tissue pulsed-wave Doppler velocity profiles | - Silhouette criterion: 0.58 | - No nocturnal blood pressure measurements  - Patients with disorder like AF were excluded  - None of patients had LV dysfunction |
| Rauseo et al.^44^ | 2026 | Machine learning | Clustering | Unsupervised | PCA and K-means | - Clinical profiles and validated on cardiac MR features | - Silhouette criterion: 0.088 | - Dominant White ethnicity  - Blood biomarkers were obtained years before the imaging  - No external testing |
| Yang et al.^41^ | 2024 | Deep learning | Clustering | Supervised | U-Net, GAN | - Cognitive, clinical, CSF volume, and plasma  - Genetic data  - Image-based features of brain tissues | - Accuracy: 90.0% | - MCI/AD were derived from small sample size  - Hierarchical relationship among multiscale clustering results was missing |
| AI Task 5  Defining risk of future hypertension-related events | | | | | | | | |
| Squirrell et al.^45^ | 2024 | Deep learning | Classification | Supervised | ResNet | - Raw retinal images | - Correlation: 0.067  - MAE: 12.35 | - Single-centre study with curated data  - Self-reported medication usage |
| Giovanna et al.^46^ | 2002 |  | Classification | Supervised | ANN | - Cardiac/vascular structures  - LV mass and dimensions  - Carotid IMT  - Urinary albumin excretion | - Accuracy: 98.3%  - Risk stratification was significantly improved with imaging data relative to routine baseline data | - Less representative cohort  - Less evidence and tests on patients with low risk profile  - Basic ANN architecture |
| Viazzi et al.^47^ | 2006 |  | Classification | Supervised | ANN (NeuroSolutions) | - Cardiac/vascular structures  - Carotid IMT  - Urinary albumin excretion  - Creatinine | - Accuracy: 94%  - Correlation: 0.95 | - The network was heavily affected by a single feature, *i.e.*, creatinine  - Basic ANN architecture |
| Other additional  applications | | | | | | | | |
| Jana et al.^48^ | 2020 | Machine learning | Regression | Supervised | SVM | - Doppler ultrasound wave spectrograms  - 2-element Windkessel model | - Correlation: 0.79  - MAE: 6.59 | - Assumption of fixed cardiac output for all patients  - Dependence on the waveform of the imaging |

A total of 48 studies were included in this systematic review. MR: magnetic resonance; CT: computed tomography; SVM: support vector machine; ResNet: residual network; RNN: recurrent neural network; PCA: principal component analysis; GAN: generative adversarial network; EKM-KELM+: empirical kernel mapping-based kernel extreme learning machine plus; CNN-BiLSTIM: convolutional neural network Bi-directional long short-term memory; ANN: artificial neural network; ROI: region-of-interest; LA: left atrium; LV: left ventricular; CSF: cerebrospinal fluid; LVH: left ventricular hypertrophy; IMT: intima–media thickness; TTE: transthoracic echocardiogram; HU: Hounsfield units; NITRA: nonlinear trimodal regression analysis; DSC: Dice similarity coefficient; MCI: mild cognitive impairment; AD: Alzheimer disease; AUC: area under the curve; ROC: receiver operating characteristics.

**Table S11. Overall summary of meta-analysis forest plots and summary operating characteristics (SROC) curves.**

| Subgroup | Studies | Tests | Total | TP | FN | Sensitivity (forest plot) | Sensitivity (SROC) | Specificity (SROC) | AUC (SROC) | Heterogeneity (I², 𝜏², p-value) |
| --- | --- | --- | --- | --- | --- | --- | --- | --- | --- | --- |
| Identifying hypertension vs healthy | | | | | | | | | | |
| Heart | 2 | 2 | 545 | 449 | 96 | 0.82  [0.79-0.85] | 0.83  [0.80-0.87] | 0.65  [0.61-0.69] | 0.84  [0.82-0.86] | I² = 0% 𝜏² = 0  p = 0.4483 |
| Brain | 2 | 2 | 43 | 41 | 2 | 0.95  [0.83-0.99] | 0.95  [0.88-1.00] | 0.86  [0.75-0.96] | 0.95  [0.92-0.98] | I² = 0% 𝜏² = 0 p = 0.3861 |
| Retinal microvasculature | 1 | 1 | 39 | 22 | 17 | 0.56  [0.40-0.72] | 0.56  [0.41-0.72] | 0.68  [0.51-0.85] | 0.66  [0.60-0.71] | NA |
| Overall | 5 | 5 | 627 | 512 | 115 | 0.84 [0.69-0.93] | 0.84 [0.81-0.87] | 0.72 [0.68-0.76] | 0.84 [0.79-0.89] | I² = 79.2% 𝜏² = 0.6643 p = 0.0007 |
| Discriminating hypertension vs hypertrophic cardiomyopathy | | | | | | | | | | |
| Overall | 9 | 9 | 521 | 481 | 40 | 0.92 [0.90-0.94] | 0.91 [0.89-0.94] | 0.81 [0.77-0.85] | 0.93 [0.91-0.95] | I² = 0% 𝜏² = 0 p = 0.7828 |

A total number of 5 studies were included in the first test (identifying hypertension vs healthy), whereas a total number of 9 studies were included in the second test (discriminating hypertension vs hypertrophic cardiomyopathy). The significance of heterogeneity was assessed using Cochran’s Q test, with *p* < 0.05 indicating statistically significant heterogeneity. NA indicates that heterogeneity analysis was not performed if only a single study was available. TP: true positive; FN: false negative; SROC: summary receiver operating characteristics. AUC: area under the curve.

**REFERENCES**

1. Morales, S., Naranjo, V., Navea, A. & Alcañiz, M. Computer-aided diagnosis software for hypertensive risk determination through fundus image processing. *IEEE J. Biomed. Health Inform.* 18, 1757–1763 (2014).

2. Lo, Y. *et al.* AML-Net: A Preliminary Screening Model for Mild Hypertension. *Proceedings - 2021 14th International Congress on Image and Signal Processing, BioMedical Engineering and Informatics, CISP-BMEI 2021* https://doi.org/10.1109/CISP-BMEI53629.2021.9624382 (2021) doi:10.1109/CISP-BMEI53629.2021.9624382.

3. Yu, X. *et al.* Analyzing brain structural differences associated with categories of blood pressure in adults using empirical kernel mapping-based kernel ELM. *Biomed. Eng. Online* 18, (2019).

4. Kandil, H. *et al.* A CAD System for the Early Prediction of Hypertension based on Changes in Cerebral Vasculature. *IST 2019 - IEEE International Conference on Imaging Systems and Techniques, Proceedings* https://doi.org/10.1109/IST48021.2019.9010179 (2019) doi:10.1109/IST48021.2019.9010179.

5. Kandil, H. *et al.* A novel computer-aided diagnosis system for the early detection of hypertension based on cerebrovascular alterations. *Neuroimage Clin.* 25, 102107 (2020).

6. Kandil, H. *et al.* Analysis of the Importance of Systolic Blood Pressure Versus Diastolic Blood Pressure in Diagnosing Hypertension: MRA Study. *Proceedings - International Conference on Image Processing, ICIP* 2020-October, 443–447 (2020).

7. Peng, B., Yu, X., Ma, X., Zhu, J. & Dai, Y. Self-paced learning based multi-kernel KRR for brain structure analysis in patients with different blood pressure levels. *Proceedings - 2020 13th International Congress on Image and Signal Processing, BioMedical Engineering and Informatics, CISP-BMEI 2020* 169–174 (2020) doi:10.1109/CISP-BMEI51763.2020.9263541.

8. Cetin, I. *et al.* A radiomics approach to analyze cardiac alterations in hypertension. *Proceedings - International Symposium on Biomedical Imaging* 2019-April, 640–643 (2019).

9. Wang, Y. *et al.* Artificial intelligence measuring the aortic diameter assist in identifying adverse blood pressure status including masked hypertension. *Postgrad. Med.* 134, 111–121 (2022).

10. Karki, S., Ali, A. E., Alsadoon, O. H. & Rashid, T. A. A novel solution of an enhanced error and loss function using deep learning for hypertension classification in traditional medicine. *CITISIA 2020 - IEEE Conference on Innovative Technologies in Intelligent Systems and Industrial Applications, Proceedings* https://doi.org/10.1109/CITISIA50690.2020.9371809 (2020) doi:10.1109/CITISIA50690.2020.9371809.

11. Rose, B., Kavya, S., Rachana, S. & Manisha, E. Cardiovascular Disease Prediction from Retinal Images using Machine Learning. *2nd International Conference on Sustainable Computing and Data Communication Systems, ICSCDS 2023 - Proceedings* https://doi.org/10.1109/ICSCDS56580.2023.10104816 (2023) doi:10.1109/ICSCDS56580.2023.10104816.

12. Koduri, H. & Ma, M. Diabetic and Hypertensive Retinopathy Classification from Retinal Images Using Dual Vision Transformer. *Proceedings - 2024 4th International Conference on Digital Data Processing, DDP 2024* 87–91 (2024) doi:10.1109/DDP64453.2024.00034.

13. Azhari, H., Gath, I., Beyar, R., Marcus, M. L. & Sideman, S. Discrimination Between Healthy and Diseased Hearts by Spectral Decomposition of Their Left Ventricular Three-Dimensional Geometry. *IEEE Trans. Med. Imaging* 10, 207–215 (1991).

14. Neisius, U. *et al.* Radiomic Analysis of Myocardial Native T1 Imaging Discriminates Between Hypertensive Heart Disease and Hypertrophic Cardiomyopathy. *JACC Cardiovasc. Imaging* 12, 1946–1954 (2019).

15. Cetin, I. *et al.* Radiomics Signatures of Cardiovascular Risk Factors in Cardiac MRI: Results From the UK Biobank. *Front. Cardiovasc. Med.* 7, 591368 (2020).

16. Vidal-Sospedra, I. *et al.* Determination of Image-based Biomarkers for the Diagnosis of Hypertrophic Cardiomyopathy, Hypertensive Cardiomyopathy and Amyloidosis from Texture Analysis in Cardiac MRI. *Proceedings - IEEE 20th International Conference on Bioinformatics and Bioengineering, BIBE 2020* 230–235 (2020) doi:10.1109/BIBE50027.2020.00045.

17. Yu, F. *et al.* Artificial intelligence-based myocardial texture analysis in etiological differentiation of left ventricular hypertrophy. *Ann. Transl. Med.* 9, 108–108 (2021).

18. Sabovčik, F. *et al.* Applying machine learning to detect early stages of cardiac remodelling and dysfunction. *Eur. Heart J. Cardiovasc. Imaging* 22, 1208–1217 (2021).

19. Shi, R. Y. *et al.* Texture analysis applied in T1 maps and extracellular volume obtained using cardiac MRI in the diagnosis of hypertrophic cardiomyopathy and hypertensive heart disease compared with normal controls. *Clin. Radiol.* 76, 236.e9-236.e19 (2021).

20. Forghani, Y., Behnam, H. & Shojaeifard, M. Hypertrophic cardiomyopathy (HCM) and hypertensive heart disease (HHD) diagnosis using echocardiography and electrocardiography. *Comput. Methods Biomech. Biomed. Eng. Imaging Vis.* 9, 565–573 (2021).

21. Barbieri, A. *et al.* Three-Dimensional Automated, Machine-Learning-Based Left Heart Chamber Metrics: Associations with Prevalent Vascular Risk Factors and Cardiovascular Diseases. *J. Clin. Med.* 11, 7363 (2022).

22. Hwang, I. C. *et al.* Differential diagnosis of common etiologies of left ventricular hypertrophy using a hybrid CNN-LSTM model. *Scientific Reports 2022 12:1* 12, 1–12 (2022).

23. Xu, Z., Yu, F., Zhang, B. & Zhang, Q. Intelligent diagnosis of left ventricular hypertrophy using transthoracic echocardiography videos. *Comput. Methods Programs Biomed.* 226, 107182 (2022).

24. Zhang, X. *et al.* Deep learn-based computer-assisted transthoracic echocardiography: approach to the diagnosis of cardiac amyloidosis. *International Journal of Cardiovascular Imaging* 39, 955–965 (2023).

25. Diao, K. *et al.* Multi-channel deep learning model-based myocardial spatial–temporal morphology feature on cardiac MRI cine images diagnoses the cause of LVH. *Insights Imaging* 14, 1–11 (2023).

26. Wang, Z. C. *et al.* Deep Learning for Discrimination of Hypertrophic Cardiomyopathy and Hypertensive Heart Disease on MRI Native T1 Maps. *Journal of Magnetic Resonance Imaging* 59, 837–848 (2024).

27. Wang, Y. R. *et al.* Screening and diagnosis of cardiovascular disease using artificial intelligence-enabled cardiac magnetic resonance imaging. *Nature Medicine 2024 30:5* 30, 1471–1480 (2024).

28. Moon, I. *et al.* Artificial Intelligence-Enhanced Analysis of Echocardiography-Based Radiomic Features for Myocardial Hypertrophy Detection and Etiology Differentiation. *Circ. Cardiovasc. Imaging* 18, e017436 (2025).

29. Recenti, M. *et al.* Healthy Aging within an Image: Using Muscle Radiodensitometry and Lifestyle Factors to Predict Diabetes and Hypertension. *IEEE J. Biomed. Health Inform.* 25, 2103–2112 (2021).

30. Kaupp, A. *et al.* Measuring morphologic properties of the human retinal vessel system using a two-stage image processing approach. *Proceedings - International Conference on Image Processing, ICIP* 1, 431–435 (1994).

31. Khitran, S., Akram, M. U., Usman, A. & Yasin, U. Automated system for the detection of hypertensive retinopathy. *2014 4th International Conference on Image Processing Theory, Tools and Applications, IPTA 2014* https://doi.org/10.1109/IPTA.2014.7001984 (2015) doi:10.1109/IPTA.2014.7001984.

32. Ahmad, F., Khan Sial, M. R., Yousaf, A. & Khan, F. Textural and Intensity Feature Based Retinal Vessels Classification for the Identification of Hypertensive Retinopathy. *Proceedings of the 21st International Multi Topic Conference, INMIC 2018* https://doi.org/10.1109/INMIC.2018.8595667 (2018) doi:10.1109/INMIC.2018.8595667.

33. Kiruthika, M., Swapna, T. R., Santhosh, K. C. & Peeyush, K. P. Artery and vein classification for hypertensive retinopathy. *Proceedings of the International Conference on Trends in Electronics and Informatics, ICOEI 2019* 244–248 (2019) doi:10.1109/ICOEI.2019.8862719.

34. Dai, G. *et al.* Exploring the effect of hypertension on retinal microvasculature using deep learning on East Asian population. *PLoS One* 15, e0230111 (2020).

35. Bhimavarapu, U., Chintalapudi, N. & Battineni, G. Automatic Detection and Classification of Hypertensive Retinopathy with Improved Convolution Neural Network and Improved SVM. *Bioengineering 2024, Vol. 11, Page 56* 11, 56 (2024).

36. Triwijoyo, B. K., Adil, A. & Zulfikri, M. Detection and classification of hypertensive retinopathy based on retinal image analysis using a deep learning approach. *Computer Methods and Programs in Biomedicine Update* 7, 100191 (2025).

37. Raghavendra, U. *et al.* Automated Diagnosis and Assessment of Cardiac Structural Alteration in Hypertension Ultrasound Images. *Contrast Media Mol. Imaging* 2022, (2022).

38. Alsharqi, M. *et al.* A machine learning-based score for precise echocardiographic assessment of cardiac remodelling in hypertensive young adults. *European Heart Journal - Imaging Methods and Practice* 1, (2023).

39. Pessana, F., Venialgo, E., Rubstein, J. & Furfaro, A. Assessment of human instantaneous arterial diameter using Bmode ultrasound imaging and Artificial Neural Networks: Determination of wall mechanical properties. *2010 Annual International Conference of the IEEE Engineering in Medicine and Biology Society, EMBC’10* 1409–1412 (2010) doi:10.1109/IEMBS.2010.5626719.

40. Alkhodari, M. *et al.* HyperScore: A unified measure to model hypertension progression using multi-modality measurements and semi-supervised learning. *Proceedings - 2023 2023 IEEE International Conference on Bioinformatics and Biomedicine, BIBM 2023* 1886–1889 (2023) doi:10.1109/BIBM58861.2023.10385558.

41. Yang, Z. *et al.* Gene-SGAN: discovering disease subtypes with imaging and genetic signatures via multi-view weakly-supervised deep clustering. *Nature Communications 2024 15:1* 15, 1–16 (2024).

42. Katz, D. H. *et al.* Phenomapping for the Identification of Hypertensive Patients with the Myocardial Substrate for Heart Failure with Preserved Ejection Fraction. *J. Cardiovasc. Transl. Res.* 10, 275–284 (2017).

43. Loncaric, F. *et al.* Automated Pattern Recognition in Whole-Cardiac Cycle Echocardiographic Data: Capturing Functional Phenotypes with Machine Learning. *Journal of the American Society of Echocardiography* 34, 1170–1183 (2021).

44. Rauseo, E. *et al.* Clinical Phenotypes in Hypertension: A Data-Driven Approach to Risk Stratification. *Hypertension* https://doi.org/10.1161/HYPERTENSIONAHA.125.25187 (2025) doi:10.1161/HYPERTENSIONAHA.125.25187.

45. Squirrell, D. M. *et al.* Blood Pressure Predicted From Artificial Intelligence Analysis of Retinal Images Correlates With Future Cardiovascular Events. *JACC: Advances* 3, 101410 (2024).

46. Leoncini, G. *et al.* Microalbuminuria identifies overall cardiovascular risk in essential hypertension: an artificial neural network-based approach. *J. Hypertens.* 20, 1315–1321 (2002).

47. Viazzi, F. *et al.* Predicting cardiovascular risk using creatinine clearance and an artificial neural network in primary hypertension. *J. Hypertens.* 24, 1281–1286 (2006).

48. Jana, B., Oswal, K., Mitra, S., Saha, G. & Banerjee, S. Windkessel Model-Based Cuffless Blood Pressure Estimation Using Continuous Wave Doppler Ultrasound System. *IEEE Sens. J.* 20, 9989–9999 (2020).
